# Analysis of tripartite Synaptotagmin‐1‐SNARE‐complexin‐1 complexes in solution

**DOI:** 10.1002/2211-5463.13503

**Published:** 2022-11-16

**Authors:** Klaudia Jaczynska, Luis Esquivies, Richard A. Pfuetzner, Baris Alten, Kyle D. Brewer, Qiangjun Zhou, Ege T. Kavalali, Axel T. Brunger, Josep Rizo

**Affiliations:** ^1^ Department of Biophysics University of Texas Southwestern Medical Center Dallas TX USA; ^2^ Department of Biochemistry University of Texas Southwestern Medical Center Dallas TX USA; ^3^ Department of Pharmacology University of Texas Southwestern Medical Center Dallas TX USA; ^4^ Department of Molecular and Cellular Physiology Stanford University CA USA; ^5^ Department of Neurology and Neurological Sciences Stanford University CA USA; ^6^ Department of Structural Biology Stanford University CA USA; ^7^ Department of Photon Science Stanford University CA USA; ^8^ Howard Hughes Medical Institute Stanford University CA USA; ^9^ Department of Pharmacology Vanderbilt University Nashville TN USA; ^10^ Vanderbilt Brain Institute Vanderbilt University Nashville TN USA; ^11^ Department of Cell and Developmental Biology Vanderbilt University Nashville TN USA; ^12^ Present address: Department of Neurology Massachusetts General Hospital Boston MA USA; ^13^ Present address: Department of Neurology Brigham and Women's Hospital Boston MA USA; ^14^ Present address: Harvard Medical School Boston MA USA; ^15^ Present address: ETTA Biotechnology Palo Alto CA USA

**Keywords:** Ca^2+^ sensing, complexin, neurotransmitter release, SNAREs, synaptotagmin, weak protein interactions

## Abstract

Characterizing interactions of Synaptotagmin‐1 with the SNARE complex is crucial to understand the mechanism of neurotransmitter release. X‐ray crystallography revealed how the Synaptotagmin‐1 C_2_B domain binds to the SNARE complex through a so‐called primary interface and to a complexin‐1‐SNARE complex through a so‐called tripartite interface. Mutagenesis and electrophysiology supported the functional relevance of both interfaces, and extensive additional data validated the primary interface. However, ITC evidence suggesting that binding via the tripartite interface occurs in solution was called into question by subsequent NMR data. Here, we describe joint efforts to address this apparent contradiction. Using the same ITC approach with the same C_2_B domain mutant used previously (C_2_B^KA‐Q^) but including ion exchange chromatography to purify it, which is crucial to remove polyacidic contaminants, we were unable to observe the substantial endothermic ITC signal that was previously attributed to binding of this mutant to the complexin‐1‐SNARE complex through the tripartite interface. We were also unable to detect substantial populations of the tripartite interface in NMR analyses of the ITC samples or in measurements of paramagnetic relaxation effects, despite the high sensitivity of this method to detect weak protein complexes. However, these experiments do not rule out the possibility of very low affinity (*K*_D_ > 1 mm) binding through this interface. These results emphasize the need to develop methods to characterize the structure of synaptotagmin‐1‐SNARE complexes between two membranes and to perform further structure–function analyses to establish the physiological relevance of the tripartite interface.

## Graphical abstract

Contradictory data were reported regarding the binding of the neuronal SNARE complex involved in neurotransmitter release to the Ca^2+^ sensor Synaptotagmin‐1 through a so‐called tripartite interface. Using ITC and highly sensitive NMR methods, we were unable to detect this interaction in solution, indicating that establishing the physiological relevance of the tripartite interface will require further research.

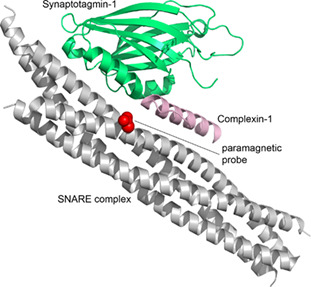

**Abbreviations**
1D: one dimensional
2D: two dimensional
FRET: fluorescence resonance energy transfer
HMQC: heteronuclear multiple quantum coherence
ITC: isothermal titration calorimetry
MD: molecular dynamics
MTSL: (1‐oxyl‐2,2,5,5‐tetramethyl‐3‐pyrroline‐3‐methyl)methanethiosulfonate
NMR: nuclear magnetic resonance
NSF: N‐ethylmaleimide sensitive factor
PIP_2_
: phosphatidylinositol 4,5‐bisphosphate
PRE: paramagnetic relaxation enhancement
SNAP: soluble NSF attachment protein
SNAP‐25: synaptosomal associated protein 25 kDa
SNARE: SNAP receptor
SNC: SNAP‐25 C‐terminal SNARE motif
SNN: SNAP‐25 N‐terminal SNARE motif
Syt1: Synaptotagmin‐1
TM: transmembrane
TROSY: transverse relaxation optimized spectroscopy
WT: wild type

The release of neurotransmitters by Ca^2+^‐evoked synaptic vesicle exocytosis is an exquisitely regulated process that is crucial for communication between neurons. Exocytosis involves several steps, which include tethering of synaptic vesicles to specialized sites of the presynaptic plasma membrane called active zones, priming of the vesicle to a state(s) that is ready for release, and very fast (< 0.1 ms) fusion of the vesicle and plasma membranes upon Ca^2+^ influx [1]. Extensive characterization of the complex protein machinery that controls these steps has allowed the reconstitution of basic features of synaptic vesicle fusion with its central components [2, 3, 4, 5] and has defined their functions (reviewed in refs [6, 7, 8]). The SNARE proteins syntaxin‐1, SNAP‐25, and synaptobrevin form a tight complex [9] that consists of a parallel four‐helix bundle [10] and brings the membranes together [11], which is critical for membrane fusion. NSF and SNAPs disassemble the SNARE complex after fusion to recycle the SNAREs [9, 12] while Munc18‐1 and Munc13s organize SNARE complex assembly by a pathway that is resistant to NSF‐SNAPs [3, 13], ensuring that the SNARE complex assembles in parallel [4]. This pathway starts with Munc18‐1 bound to a self‐inhibited ‘closed’ conformation of syntaxin‐1 [14, 15]. Munc18‐1 later binds to synaptobrevin, forming a template for SNARE complex assembly [16, 17, 18, 19], whereas Munc13‐1 helps open syntaxin‐1 [20, 21, 22] while bridging the vesicle and plasma membranes [23, 24] and promoting parallel assembly of SNAP‐25 with syntaxin‐1 and synaptobrevin in conjunction with Munc18‐1 [4]. Synaptotagmin‐1 (Syt1) (as well as its homologs Syt2 and Syt9) acts as the Ca^2+^ sensor that triggers fast neurotransmitter release [25] in a tight interplay with complexins [26, 27, 28] involving the formation of a spring‐loaded Syt1‐SNARE‐complexin macromolecular assembly [29, 30, 31, 32] that most likely constitutes a central element of primed synaptic vesicles, keeping the system ready for fast Ca^2+^‐triggered fusion but preventing premature fusion before Ca^2+^ influx [31, 33, 34]. Despite these advances, the final steps leading to fast Ca^2+^‐triggered membrane fusion remain unclear, in part because it has been challenging to elucidate how the functions of Syt1, complexins, and the SNAREs are coupled [6, 7].

Complexins are small soluble proteins that bind tightly to the SNARE complex [35] and play both stimulatory and inhibitory roles in release [36, 37]. The crystal structure of a complexin‐1‐SNARE complex showed that SNARE binding is mediated by a central helix of complexin‐1 that is preceded by a so‐called accessory helix [29]. The central helix is critical for all complexin functions while the accessory helix is responsible at least in part for the inhibitory activity [38, 39], likely because it hinders C‐terminal zippering of the SNARE complex due to steric clashes with the vesicle [34, 40] and/or interactions with the SNAREs [41, 42]. N‐ and C‐terminal sequences of complexins also contribute to their dual functions through interactions with the lipids and perhaps with the SNAREs [41, 43, 44, 45, 46, 47].

Syt1 is a synaptic vesicle protein with two C_2_ domains (termed C_2_A and C_2_B) that form most of its cytoplasmic region and bind three and two Ca^2+^ ions, respectively, through loops at the tip of β‐sandwich structures [48, 49, 50]. These loops also bind to membranes in a Ca^2+^‐dependent manner, which is crucial for the function of Syt1 in triggering release [25, 51] and is mediated in part by interactions of basic residues from these loops with negatively charged phospholipids [52]. The Syt1 C_2_B domain can also bind to membranes through two additional areas that are rich in basic residues (Fig. 1A) and can also interact with the SNAREs, which have abundant negative charges. Thus, a polybasic region at one side of the C_2_B domain β‐sandwich binds to PIP_2_, which was proposed to steer Syt1 to the plasma membrane [53], but NMR studies showed that the polybasic region also binds to the center of SNARE complex four‐helix bundle [54] (Fig. 1B) (please note that here we use the terms polybasic and poliacidic, which are widely employed in the protein literature, to refer to the more accurate terms polycationic and polyanionic, respectively). The clear correlation of the disruptive effects of mutations in the polybasic region on SNARE complex binding with those caused by neurotransmitter release supported the physiological relevance of this binding mode [54], but it was later shown that the physiological effects of these mutations most likely arise because of disruption of Ca^2+^‐dependent interactions of the Syt1 C_2_B domain with PIP_2_ on the plasma membrane [33]. It is also worth noting that the C_2_B domain polybasic region was also implicated in interactions with the C‐terminus of membrane‐anchored SNARE complexes that compete with the binding of these complexes to a complexin‐1 fragment [55], even though the same fragment and Syt1 can bind simultaneously to soluble SNARE complexes [56]. These findings underline the complexity of deciphering the functional interplay between Syt1 and complexins.

**Fig. 1:**
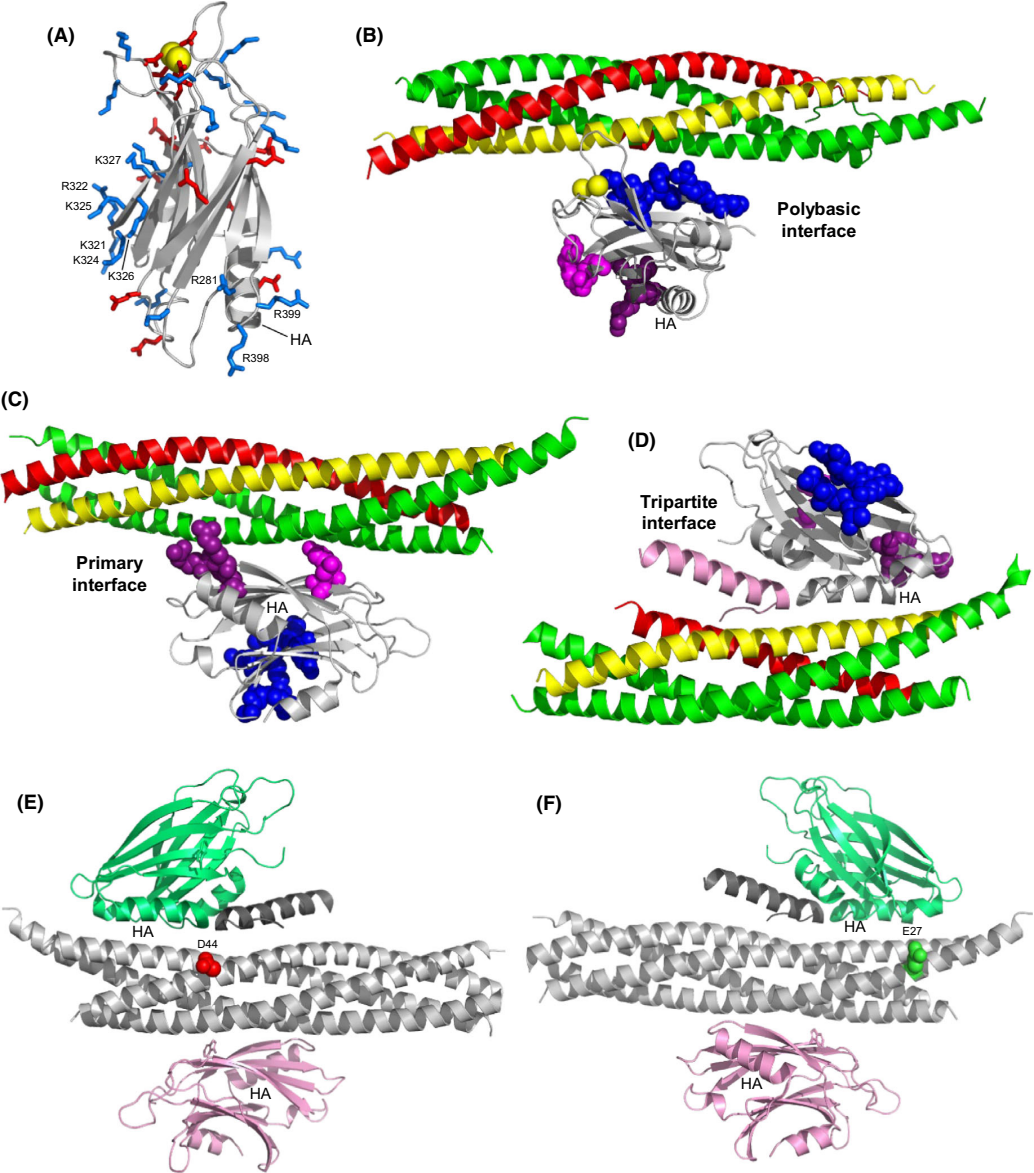
Structures of Syt1‐SNARE complexes. (A) Ribbon diagram of the NMR structure of the Syt1 C_2_B (B) domain [50] (PDB accession number 1K5W) showing acidic and basic side chains as stick models colored in red and blue, respectively. Selected residues discussed in the text are indicated. Helix A is labeled as HA. Bound Ca^2+^ ions are shown as yellow spheres. (B–D) ribbon diagrams of a representative conformer of the dynamic C_2_B domain‐SNARE complex structure determined in solution by NMR spectroscopy [54] (B), of a C_2_B domain‐SNARE complex structure determined by X‐ray crystallography [30] (C), and of a C_2_B domain‐SNARE‐complexin‐1 complex structure determined also by X‐ray crystallography [31] (D). The PDB accession numbers are 2N1T, 5KJ7 and 5W5C, respectively. The Syt1 C_2_B domain is in light gray, complexin‐1 in pink, synaptobrevin in red, syntaxin‐1 in yellow and SNAP‐25 in green. Residues from the C_2_B polybasic region are shown as blue spheres and selected C_2_B residues that form the primary are shown as magenta (E295 and Y338) or deep purple (R281, R398, and R399) spheres. (E, F) ribbon diagrams showing the SNARE complex in light gray, complexin‐1 in dark gray, and two copies of the C_2_B domain bound through the primary interface (pink) or the tripartite interface (lime green). The diagrams are in two different orientations to show the locations of residues that were mutated to cysteine for labeling with MTSL, namely residue D44 of synaptobrevin (red spheres) (E) and residue E27 of SNAP‐25 (green spheres) (F).

At the tip of the β‐sandwich opposite to the Ca^2+^‐binding loops, the C_2_B domain contains an arginine‐rich region that can also interact with membranes and mediate membrane‐membrane bridging when the Ca^2+^‐binding loops bind simultaneously to another membrane, an activity that was proposed to cooperate with the SNAREs in bringing the vesicle and plasma membranes together [57, 58]. This proposal was supported by the strong disruption of neurotransmitter release caused by R398Q and R399Q mutations in this region [59]. However, crystal structures of Syt1‐SNARE and Syt1‐SNARE‐complexin‐1 complexes showed that R398 and R399 participate in the binding of the Syt1 C_2_B domain to the SNARE complex through the so‐called primary interface [30, 31] (Fig. 1C), and there is now overwhelming evidence supporting the physiological relevance of this binding mode [30, 32, 33, 60].

A Syt1‐SNARE‐complexin‐1 crystal structure revealed yet another binding mode in which an α‐helix of the C_2_B domain binds to the SNARE complex, continuing the complexin‐1 central helix [31] (Fig. 1D). The surface involved in this binding mode is referred to as the tripartite interface. ITC experiments using SNARE complex and Syt1 C_2_B domain‐bearing mutations to disrupt binding through the primary interface and the polybasic region supported the existence of this binding mode in solution and indicated that complexin‐1 is required for Syt1‐SNARE complex binding through this mode. Moreover, the disruption in binding and in neurotransmitter release caused by a mutation in the tripartite interface and the requirement of complexin‐1 for the rescue of dominant negative Ca^2+^‐binding mutations of the Syt1 C_2_B domain supported the physiological relevance of this interface [31]. However, no binding of the Syt1 C_2_B domain to a complexin‐1‐SNARE complex through the tripartite interface was detected using NMR spectroscopy even when a different set of mutations in the C_2_B domain were used to abrogate the interactions involving the primary interface and the polybasic region [33].

Clarifying these apparently contradictory data in our laboratories is critical to assess the physiological relevance of the tripartite interface and understand how Ca^2+^ influx is coupled to membrane fusion during neurotransmitter release. With this goal, we have joined forces and have further analyzed tripartite Syt1‐SNARE‐complexin‐1 complexes in solution using a combination of NMR spectroscopy and ITC. We could not obtain definitive evidence for binding of the Syt1 C_2_B domain to complexin‐1‐ SNARE complexes through the tripartite interface in solution, but we cannot rule out the possibility that such binding might be strengthened by co‐localization and/or cooperativity with Syt1‐membrane interactions *in vivo*. Overall, our results emphasize the need to develop tools to study Syt1‐SNARE‐complexin complexes between two membranes, mimicking the environment experienced by these proteins in the primed state of synaptic vesicles.

## Materials and methods

### Protein expression and purification

**Fig. 2:**
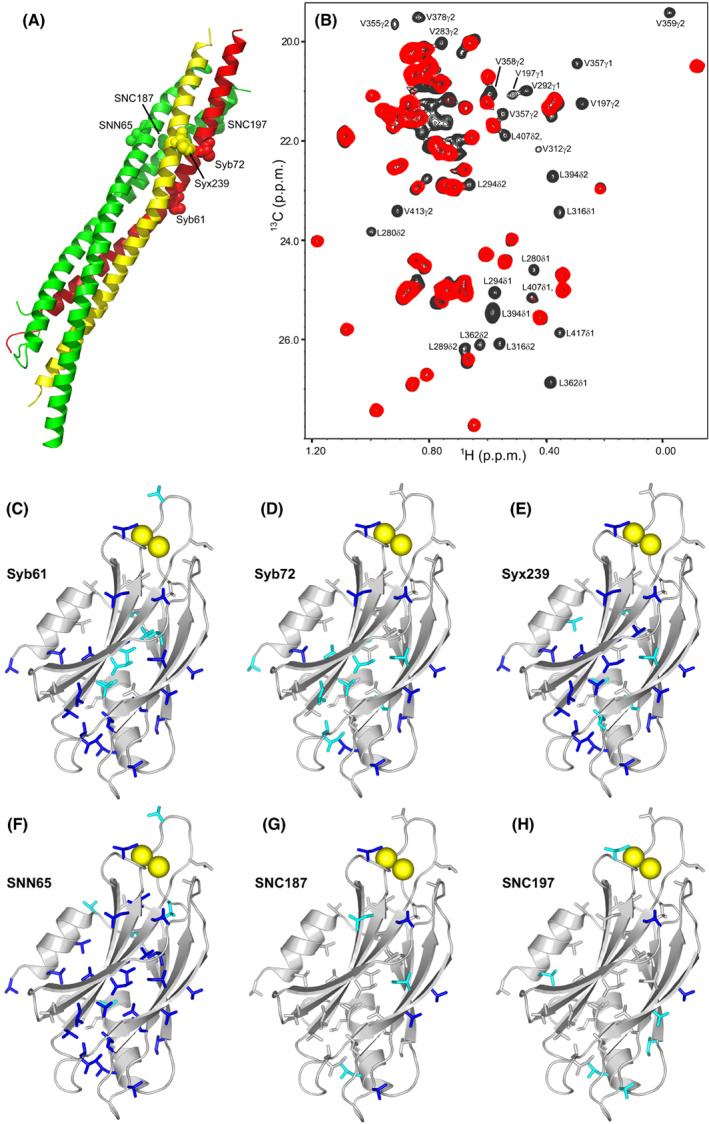
PRE analysis of Syt1 C_2_AB binding to the C‐terminal half of the SNARE complex. (A) Ribbon diagram of the SNARE complex (synaptobrevin in red, syntaxin‐1 in yellow, and SNAP‐25 in green) showing the locations of residues that were mutated to cysteine for labeling with MTSL. SNN and SNC refer to the N‐ and C‐terminal SNARE motifs of SNAP‐25, respectively. (B) ^1^H‐^13^C HMQC spectra of ^15^ N‐^2^H–ILV‐^13^CH_3_–labeled Syt1 C_2_AB in the presence of SNARE complex bearing an MTSL probe on residue 65 of SNAP‐25 before (red contours) and after (black contours) reduction of the MTSL probe. The residues corresponding to selected cross‐peaks exhibiting strong PREs are labeled. (C–F) ribbon diagrams of the Syt1 C_2_B domain (light gray) showing the methyl‐containing side chains that exhibit PREs smaller than 0.4 as blue sticks and those exhibiting PREs between 0.4 and 0.6 as cyan sticks in ^1^H‐^13^C HMQC experiments performed with ^15^ N‐^2^H–ILV‐^13^CH_3_–labeled Syt1 C_2_AB and SNARE complex tagged with MTSL at residue 61 or 72 of synaptobrevin (Syb61 and Syb72, respectively) (C, D), residue 239 of syntaxin‐1 (Syx239) (E), or residue 65, 187 or 197 of SNAP‐25 (SNN65, SNC187, and SNC197, respectively) (F–H). Bound Ca^2+^ ions are shown as yellow spheres for illustrative purposes, but note that the experiments were performed in the absence of Ca^2+^.

For the PRE experiments of Fig. 2, we used pGEX‐KT constructs that encode rat synaptobrevin‐2 residues 29–93, rat syntaxin‐1A residues 191–253, human SNAP‐25A residues 11–82 and human SNAP‐25A residues 141–203, as well as a pGEX‐KG construct that encodes rat synaptotagmin‐1 residues 140–421 (C_2_AB). These constructs, as well as the procedures to express the proteins as GST fusions in *E. coli* BL21 (DE3) cells and to purify them, were described previously [29, 57]. The following single cysteine SNARE mutants for attachment of MTSL were generated using the QuickChange site‐directed mutagenesis kit (Stratagene, Sacramento, CA, USA): synaptobrevin‐2(29–93) S61C, synaptobrevin‐2(29–93) A72C, syntaxin‐1A (191–253) H239C, SNAP‐25 (11–82) N65C, SNAP‐25 (141–203) S187C and SNAP‐25 (141–203) Q197C. The mutants were expressed and purified by the same protocols as the WT proteins. ^15^ N,^2^H–labeled Syt1 C_2_AB specifically ^1^H‐^13^C‐labeled at isoleucine, leucine, and valine methyl groups (^15^ N‐^2^H–ILV‐^13^CH_3_–labeled) was obtained by expression in M9 minimal medium in 99.9% D_2_O with d‐glucose (1,2,3,4,5,6,6‐D_7_, 97–98%) as the sole carbon source (3 g·L^−1^) and ^15^NH_4_Cl as the sole nitrogen source (1 g·L^−1^), adding [3,3‐^2^H_2_]^13^C‐methyl alpha‐ketobutyric acid (80 mg·L^−1^) and [3‐^2^H]^13^C‐dimethyl alpha‐ketoisovaleric acid (80 mg·L^−1^) (Cambridge Isotope Laboratories, Tewksbury, MA, USA) to the cell cultures 30 min before isopropyl β‐d‐1‐thiogalactopyranoside (IPTG) induction.

**Fig. 3:**
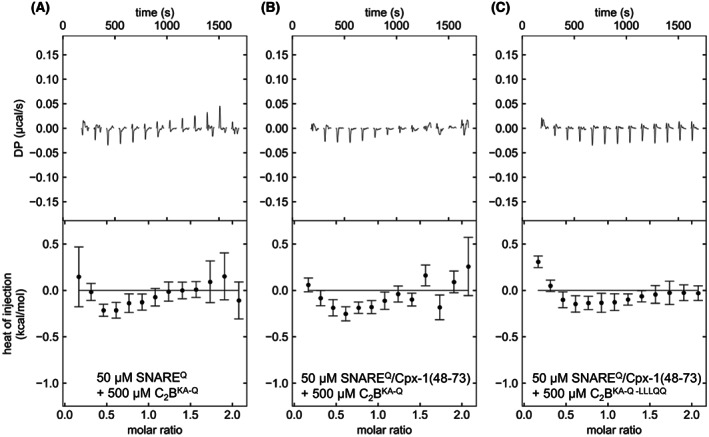
ITC analysis of interactions between C_2_B^KA‐Q^ and SNARE^Q^. The panels show titrations of 50 μm SNARE^Q^ (A) or SNARE^Q^/Cpx1(48‐73) (B, C) with 500 μm C_2_B^KA‐Q^ (A, B) or C_2_B^KA‐Q‐LLQQ^ (C) monitored by ITC. The upper panels show the baseline‐ and singular‐value‐decomposition‐corrected thermograms for the respective experiments. The circles in the lower panels are the integrated heats of injection, with the error bars representing estimated errors for these values.

**Fig. 4:**
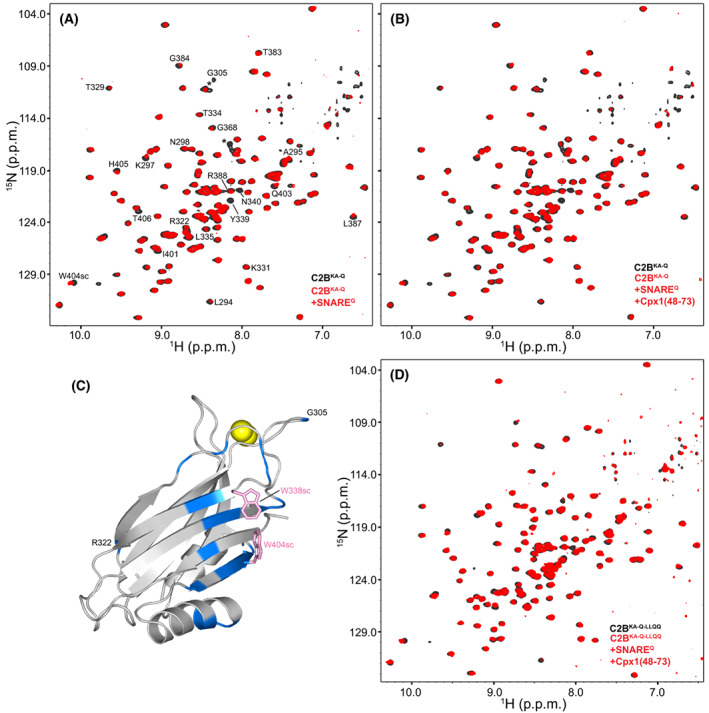
NMR analysis of the ITC samples used for the study of interactions between C_2_B^KA‐Q^ and SNARE^Q^. (A, B, D) Superpositions of ^1^H‐^15^ N TROSY‐HSQC spectra of isolated ^15^ N‐labeled C_2_B^KA‐Q^ (A, B) or C_2_B^KA‐Q‐LLQQ^ (D) (black contours) and of the final samples of ITC experiments analogous to those of Fig. 3 after titration of SNARE^Q^ (A) or SNARE^Q^/Cpx1(48‐73) (B, D) with ^15^ N‐labeled C_2_B^KA‐Q^ (A, B) or C_2_B^KA‐Q‐LLQQ^ (D) (red contours). (C) Ribbon diagram of the Syt1 C_2_B domain with the residues that exhibited strongest perturbations in ^1^H‐^15^ N TROSY‐HSQC spectra of ^15^ N‐labeled C_2_B^KA‐Q^ after titration onto SNARE^Q^ (spectra shown in panel A). The ratio between the intensities of the cross‐peaks in the spectrum of the final ITC sample containing ^15^ N‐labeled C_2_B^KA‐Q^ and SNARE^Q^ and the intensities of the cross‐peaks of isolated ^15^ N‐labeled C_2_B^KA‐Q^ were calculated. All intensities were obtained at the cross‐peak positions corresponding to isolated ^15^ N‐labeled C_2_B^KA‐Q^. Since the protein concentrations were different, the average of all the ratios was calculated and used to normalize all ratios. Residues with normalized ratios below 0.75, which result because of exchange broadening or cross‐peak shifts, are colored in blue on the ribbon diagram. The side chains of W338 and W404, which are at the center of the surface of C_2_B^KA‐Q^ that binds to SNARE^Q^ and likely contribute to binding, are shown as pink stick models. Ca^2+^ ions are shown as yellow spheres for illustrative purposes, but note that the experiments were performed in the absence of Ca^2+^.

**Fig. 5:**
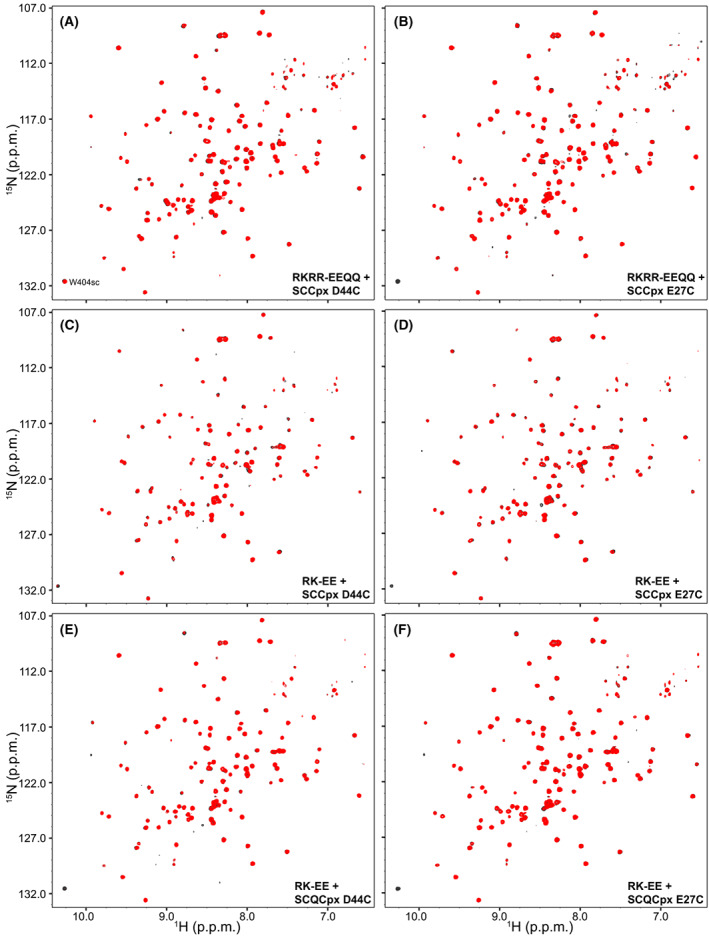
Analysis of potential tripartite Syt1‐SNARE‐complexin‐1 complexes using PREs. The panels shown ^1^H‐^15^ N TROSY‐HSQC spectra of 20 μm ^15^ N,^2^H‐labeled C_2_B^RKRR‐EEQQ^ (A, B) or ^15^ N,^2^H‐labeled C_2_B^RK‐EE^ (D–F) in the presence of 110 μm MTSL‐labeled SCCpx D44C (A, C), SCCpx E27C (B, D), SC^Q^Cpx D44C (E), or SC^Q^Cpx E27C (F) before (red contours) or after (black contours) reduction of the MTSL probe.

**Fig. 6:**
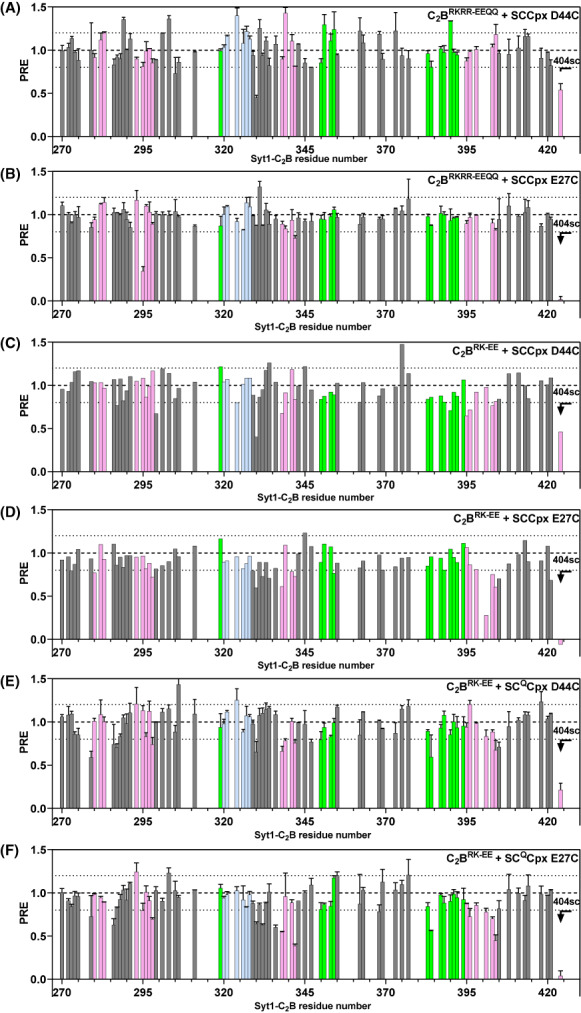
PREs observed in the analysis of potential tripartite Syt1‐SNARE‐complexin‐1 complexes. The panels show bar diagrams of PREs versus residue number that was calculated from ^1^H‐^15^ N TROSY‐HSQC spectra of 20 μm ^15^ N,^2^H‐labeled C_2_B^RKRR‐EEQQ^ (A, B) or ^15^ N,^2^H‐labeled C_2_B^RK‐EE^ (D–F) in the presence of 110 μm MTSL‐labeled SCCpx D44C (A, C), SCCpx E27C (B, D), SC^Q^Cpx D44C (E), or SC^Q^Cpx E27C (F) by dividing the intensity observed before reduction by the intensity observed after reduction. PREs observed for the W404 side chain NH group are shown on the right. The bars are colored in pink for residues from the primary interface, light blue for residues from the polybasic interface, and green for residues from the tripartite interface. Dashed lines serve as references for PRE = 0.8 and 1.2. Error bars indicate standard deviations for experiments that were performed in duplicate.

**Fig. 7:**
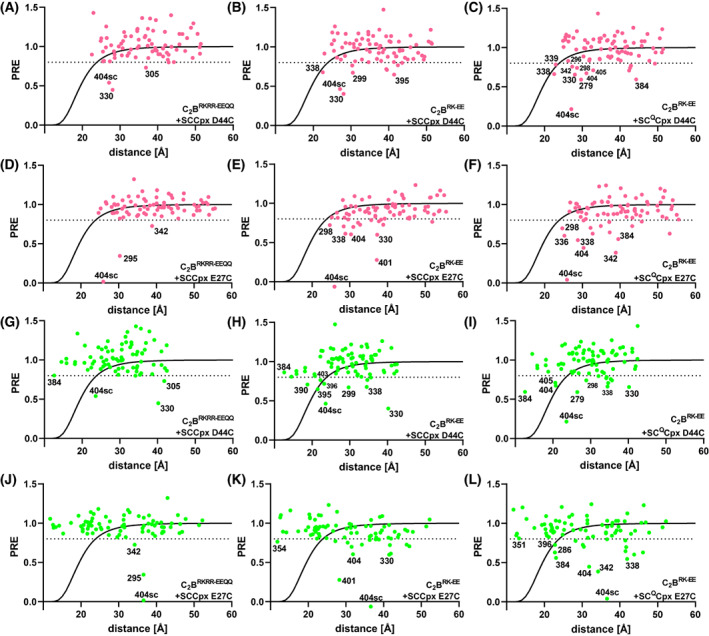
PRE analyses based on the crystal structures that revealed the primary and tripartite interfaces. The graphs show plots of the PREs observed in ^1^H‐^15^ N TROSY‐HSQC spectra of 20 μm ^15^ N,^2^H‐labeled C_2_B^RKRR‐EEQQ^ (A, D, G, J) or ^15^ N,^2^H‐labeled C_2_B^RK‐EE^ (B, C, E, F, H, I, K, L) in the presence of 110 μm MTSL‐labeled SCCpx D44C (A, B, G, H), SCCpx E27C (D, E, J, K), SC^Q^Cpx D44C (C, I), or SC^Q^Cpx E27C (F, L) versus the distance from the corresponding nitrogen atom to the estimated position of the MTSL probe predicted if binding occurs through the primary interface (A‐F) or the tripartite interface (G–L). PDB accession numbers 5kj7 and 5w5c were used to predict the distances corresponding to the primary interface and the tripartite interface, respectively. Selected PREs below 0.8 are labeled in each plot to illustrate particular points discussed in the text.

**Fig. 8:**
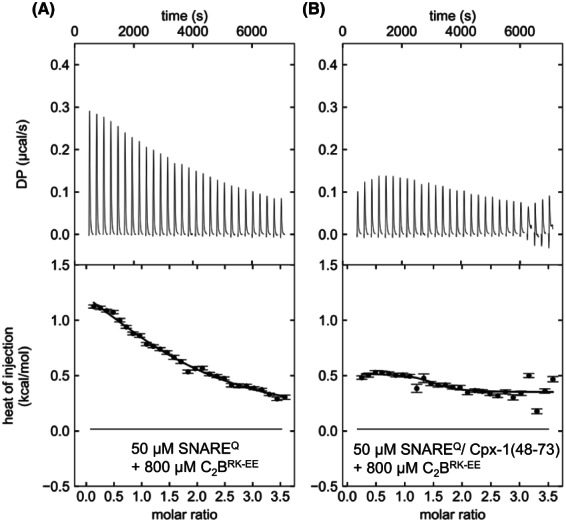
ITC analysis of interactions between C_2_B^RK‐EE^ and SNARE^Q^. The panels show titrations of 50 μm SNARE^Q^ (a) or SNARE^Q^/Cpx1(48‐73) (B) with 800 μm C_2_B^RK‐EE^ monitored by ITC. The upper panels show the baseline‐ and singular‐value‐decomposition‐corrected thermograms for the respective experiments. The circles in the lower panels are the integrated heats of injection, with the error bars representing estimated errors for these values.

The following constructs, which were described previously [29, 31, 33], were used for the ITC and PRE experiments of Figs 3, 4, 5, 6, 7, 8: Duet constructs for co‐expression of the four SNARE motifs that form the neuronal SNARE complex, encoding rat synaptobrevin‐2 residues 28–89, rat syntaxin‐1A residues 191–256, rat SNAP‐25 residues 7–83 and rat SNAP‐25 residues 140–204; the same Duet constructs but bearing 5 mutations in SNAP25 [K40A/D51A/E52A/E55A/D166A (referred to as SNARE^Q^)]; a pGEX‐6P‐1 vector to express rat complexin‐1 residue 48–73; a pETDuet construct to express rat complexin‐1 residues 26–83; pGEX‐6P‐1 constructs to express rat synaptotagmin‐1 residues 271–421 bearing the R281A/E295A/K326/K327A/Y338W/R398A/R399A mutations (referred to as C_2_B^KA‐Q^) or the R281A/E295A/K326/K327A/Y338W/L387Q/L394Q/R398A/R399A mutations (referred to as C_2_B^KA‐Q‐LLQQ^); pGEX‐KG constructs to express rat synaptotagmin‐1 residues 270–421 bearing the R322E/K325E mutations (referred to as C_2_B^RK‐EE^) or the R322E/K325E/R398Q/R399Q mutations (referred to as C_2_B^RKRR‐EEQQ^). Duet constructs for co‐expression of the four SNARE motifs but bearing the D44C mutation in synaptobrevin‐2 (28–89) or the E27C in SNAP‐25 (7–83) for MTSL tagging were prepared using the QuickChange II Site‐Directed Mutagenesis kit (Agilent, Santa Clara, CA, USA). Constructs prepared in the Brunger lab were shared with the Rizo laboratory, and vice versa, to ensure that the proteins expressed in both labs had identical amino acid sequences.

All synaptotagmin‐1 C_2_B domain mutants used in this study were expressed and purified by both laboratories following the procedures described below, following those previously reported [61]. ^15^ N labeled mutant C_2_B domains for ITC and subsequent NMR spectroscopy were expressed using M9 expression media with ^15^NH_4_Cl as the sole nitrogen source (1 g·L^−1^). ^2^H,^15^ N‐labeled mutant C_2_B domains for PRE experiments were expressed using M9 minimal medium in 99.9% D_2_O with D‐glucose (1,2,3,4,5,6,6‐D_7_, 97–98%) as the sole carbon source (3 g·L^−1^) and ^15^NH_4_Cl as the sole nitrogen source (1 g·L^−1^). The cell cultures were grown to OD ~ 0.8 at 37 °C. After adding IPTG to a concentration of 0.4 mm, the protein was expressed overnight at 25 °C. The cells were harvested by centrifugation (30 min at 5,909 *g*), and the pellet was resuspended in cold lysis buffer (40 mm Tris–HCl pH 8.2, 200 mm NaCl, 2 mm DTT) supplemented with 1% Triton and protease inhibitors (Sigma‐Aldrich, St. Louis, MO, USA). The cells were lysed using an EmulsiFlex‐C5 (Avestin) at 13,000 psi and spun at 43,667 *g* for 40 min. The supernatant was incubated for 1 h at RT with protamine sulfate, 100 mg per 1 L of culture (Sigma‐Aldrich, St. Louis, MO, USA). The mixture was spun again at 19,000 rpm for 30 min, and the supernatant was incubated overnight at 4 °C with prewashed Glutathione Sepharose 4B beads (GE Healthcare Life Sciences, Chicago, IL, USA), using 2 mL of slurry per 1 L of culture (GE Healthcare Life Science). The resin was extensively washed with 200 mL of each of the following buffers: lysis buffer, lysis buffer with 50 mm CaCl_2_, and lysis buffer with 50 mm CaCl_2_ and 1 m NaCl. The resin was then equilibrated with nuclease buffer (50 mm Tris–HCl, pH 8.0, 2 mm MgCl_2_, 2 mm DTT) followed by the addition of 10 mL of nuclease buffer and 10 μL of Universal Nuclease (Thermo Scientific, Waltham, MA, USA) and rotating the mixture at RT for 2 h. The nuclease wash was discarded, and the resin was then equilibrated with thrombin cleavage buffer (TCB: 50 mm Tris–HCl, pH 8.0, 150 mm NaCl, 2.5 mm CaCl_2_, 2 mm DTT). Thrombin cleavage was carried out at RT for 3 h or at 4 °C overnight in 10 mL of TCB and 0.08 mg·mL^−1^ thrombin (Sigma‐Aldrich) or HRV 3C Protease, depending on the construct. The cleavage fraction was collected and elution was repeated with TCB until UV Abs_280_ < 0.1 in order to recover the maximal amount of protein from the resin. The fractions containing the cleaved protein were combined and subjected to cation‐exchange chromatography using the following buffers: 20 mm MES pH 6.2, 20 mm CaCl_2_, 0.3 mm TCEP (Buffer A); and 20 mm MES pH 6.2, 20 mm CaCl_2,_ 1 m NaCl, 0.3 mm TCEP (Buffer B). The fractions eluted from the affinity column were diluted with buffer A so that [NaCl] was ≤100 mm. Cation exchange was performed on a HiTrap SP 5 mL column (GE Life Sciences) using a linear gradient from 0 to 1 m NaCl over at least 12 column volumes (> 60 mL). It should be noted that the cation‐exchange chromatography step gave at least two distinct peaks, both corresponding to protein of the correct molecular weight when run on SDS/PAGE gel. Only the later fractions contained C_2_B domain devoid of polyacidic contaminants. The previous steps (protamine sulfate and nuclease treatments) minimized the presence of polyacidic contaminants in the preparation, significantly increasing the yield of the pure fractions. Collected fractions were concentrated to 100–1200 μm depending on the next experimental steps. The concentration of all purified protein was estimated by measuring UV absorbance at 280 nm unless specified otherwise.

The quintuple mutant of the SNARE complex (SNARE^Q^) used for ITC experiments was expressed and purified as follows. BL21 (DE3) cells were co‐transformed with plasmids containing decimal‐histidine‐tagged, synaptobrevin‐2 (28–89), syntaxin‐1A (191–256), SNAP‐25 (7–83, K40A, D51A, E52A, E55A), and SNAP‐25 (191–204, D166A). Cells were grown using an auto‐inducing LB medium at 37 °C for 8 h, followed by 16 h at 30 °C. Cells were harvested by centrifugation and resuspended in lysis buffer (50 mm Sodium Phosphate pH 8, 300 mm NaCl, 10 mm Imidazole, 0.5 mm TCEP, 1% Triton X‐100) supplemented with lysozyme (Sigma), DNAse I (Sigma), and protease inhibitor tablets (Roche, Basel Switzerland). Cells were lysed by sonication and subjected to centrifugation to clear cell debris. The cleared cell lysate was mixed with Ni‐NTA agarose beads (Qiagen,) previously equilibrated with lysis buffer and incubated for 2 h at 4 °C. After incubation, the beads were harvested by centrifugation and poured into a column. The beads were subsequently washed with 8 column volumes (CV) of lysis buffer (without TX‐100), Urea wash buffer (50 mm NaPi pH 8, 300 mm NaCl, 30 mm Imidazole, 0.5 mm TCEP, 7.5 m Urea), and Wash buffer (50 mm NaPi, 30 mm Imidazole, 300 mm NaCl, 0.5 mm TCEP). The complex was eluted with wash buffer supplemented with 370 mm Imidazole. All washes, as well as the elution, were done at RT. A Bradford assay was used to detect the fractions containing the complex. These fractions were combined, supplemented with tobacco etch virus protease, and dialyzed against cleavage buffer (50 mm Tris pH 8, 250 mm NaCl, 0.5 mm TCEP, 1 mm EDTA) overnight at RT to remove the Histidine tag. After cleavage and dialysis, the NaCl concentration was adjusted to 50 mm using cleavage buffer without NaCl in preparation for anion exchange chromatography (buffer A: 50 mm Tris pH 8, 50 mm NaCl, 0.5 mm TCEP, 1 mm EDTA; buffer B: 50 mm Tris pH 8, 500 mm NaCl, 0.5 mm TCEP, 1 mm EDTA). The complex was purified using a linear NaCl gradient (50–500 mm). Peak fractions were pooled, concentrated to ~ 350 μm, flash‐frozen in liquid nitrogen, and stored at −80 °C.

Two versions of the SNARE^Q^ complex were used for the PRE NMR experiments, each having the MTSL label in a different position (D44C of synaptobrevin or E27C of SNAP‐25). The protein complex was expressed and purified in the same manner as the ones used for ITC experiments, with the addition of a final buffer exchange: after anion exchange chromatography, the pooled fractions were concentrated and loaded onto a Superdex 75 16/600 column (Cytiva, Marlborough, MA, USA) equilibrated with 50 mm Tris pH 8.0, 200 mm NaCl. After buffer exchange, the complex was flash frozen and stored at −80 °C.

### Labeling with MTSL

To label the synaptobrevin‐2 (29–93) S61C, synaptobrevin‐2 (29–93) A72C, syntaxin‐1A (191–253) H239C, SNAP‐25 (11–82) N65C, SNAP‐25 (141–203) S187C and SNAP‐25 (141–203) Q197C mutants with MTSL for the PRE experiments of Fig. 2, each protein was first treated with 10 mm DTT, and the DTT was then removed by gel filtration. The protein was concentrated to 40–60 μm and a 10‐fold excess of MTSL was added from a 40‐mm stock in dimethyl sulfoxide. The mixture was rotated overnight at 4 °C to allow complete cysteine labeling and the excess MTSL was removed by buffer exchange in 25 mm Tris pH 7.4 (pD 7.8), 125 mm NaCl and 1 mm EDTA. The MTSL‐labeled proteins were mixed with the other three SNARE motifs to form the SNARE and the unassembled fragments were removed by concentration dilution through a filter with a 10 kDa cutoff as described [29].

To label the D44C and E27C SNARE complexes for the PRE experiments of Figs 5, 6, 7, 10‐fold excess of MTSL was added to the assembled complex from a 40 mm stock in dimethyl sulfoxide and rotated overnight at 4 °C. Excess label was removed by concentrating the sample to 2–5 mL and loading it on to a Superdex 75 16/600 column equilibrated with 20 mm HEPES pH 7.4, 100 mm KCl. Peak fractions were collected and concentrated to 350–400 μm. The labeled complex was flash‐frozen and stored at −80 °C. Quantitative MTSL labeling was confirmed by Mass spectrometry.

### Isothermal calorimetry

All ITC measurements in the Rizo laboratory were performed at 25 °C on an ITC200 microcalorimeter (Microcal, GE Healthcare) except for those shown in Fig. S5H–J, which were performed on a VP‐ITC microcalorimeter (Microcal, GE Healthcare). All protein samples were dialyzed against 10 mm HEPES (pH 7.4), 100 mm NaCl, and 0.5 mm TCEP for 3 h, 5 h, and overnight using the Thermo Scientific™ Slide‐A‐Lyzer™ Dialysis Cassettes in 2 L. The final buffer was used as a washing buffer for the ITC instrument and for dilutions of the samples to the desired concentrations. Samples were degassed for 10 min before each experiment. The SNARE^Q^ complex or SNARE^Q^/Cpx(48‐73) solutions (10–50 μm) were placed in the sample cell. Solutions of Syt1 C_2_B mutants or Cpx(48‐73) (450–500 μm and 30–120 μm, respectively) were loaded into the syringe. Injection volumes for mutant Syt1 C_2_B mutants were 2.9 μL (14 injections) and for Cpx(48‐73) 1.9 μL (21 injections) on ITC200, and 20 μL (16 injections) and 10 μL (30 injections), respectively on VP‐ITC. The same concentration of mutant Syt1 C_2_B solution was injected into the buffer alone to make sure the solvents were well matched and there was no heat of injection by the buffer alone. The data were baseline corrected and integrated with NITPIC. Next, the data were fitted with a nonlinear least squares routine using a single‐site binding model with Sedphat and plotted with GUSSI [62]. The ‘A + B <−>AB’ model was used for the fitting selected data sets, and apparent concentration errors for the cell contents were compensated by refining an incompetent fraction parameter. After the titrations, 300 μL of the ITC samples were collected, supplemented with 10% D_2_O, and used for NMR spectroscopy.

ITC measurements in the Brunger laboratory were performed on a VP‐ITC microcalorimeter using the same procedures except that the pH of the buffers was 7.5, that samples were dialyzed for 3 h in 1 L of buffer, overnight in 2 L of buffer and 3 h in 1 L of buffer, and that the volume of the injections of mutant C_2_B was 10 μL instead of 20 μL.

### NMR spectroscopy

All NMR spectra were acquired at 25 °C on Agilent DD2 spectrometers equipped with cold probes operating at 600 MHz (^1^H‐^15^ N TROSY‐HSQC spectra of ITC samples and triple resonance experiments) or 800 MHz (^1^H‐^13^C HMQC spectra and ^1^H‐^15^ N TROSY‐HSQC spectra for PRE experiments). ^1^H‐^13^C HMQC spectra were acquired in D_2_O with samples containing 32 μm ^15^ N‐^2^H–ILV‐^13^CH_3_–labeled C_2_AB and 64 μm MTSL‐labeled SNARE complex and a buffer composed of 25 mm Tris pH 7.4 (pD 7.8), 125 mm NaCl and 1 mm EDTA. Spectra were acquired before and after the reduction of the MTSL probe with 1 mm sodium dithionite and 1 mm ascorbic acid (from 100‐mm stocks). ^1^H‐^15^ N TROSY‐HSQC was acquired on samples with 10% D_2_O as the solvent. In addition to the experiments performed with the ITC samples, ^1^H‐^15^ N TROSY‐HSQC spectra were acquired for C_2_B mutants alone (100 μm concentration) in dialysis buffer supplemented with 1 mm EDTA or 1 mm CaCl_2_. Experimental details of sample preparation for the ^1^H‐^15^ N HSQC spectra used for PRE measurements are described below. The acquisition times for the different spectra were 1–7 h, depending on the sample concentrations. The backbone resonances of C_2_B^KA‐Q^ were assigned using HNCACB and CBCACONH spectra acquired with sensitivity‐enhanced, pulsed field gradient sequences [63]. All NMR data were processed with NMRPipe [64] and analyzed with NMRView [65].

### PRE experiments

^15^ N,^2^H–labeled C_2_B^RK‐EE^ and C_2_B^RKRR‐EEQQ^ used for PRE experiments have native cysteine residue and were purified in the presence of the reducing agent TCEP. TCEP was removed by gel filtration using 20 mm HEPES pH 7.4100 mm KCl. As observed previously, introducing the R322E/K325E mutation increases the stability of the β‐sheet and slows down the H/D exchange rate of some amide hydrogens [33]. In order to exclude any intensity change due to H/D exchange, the C_2_B mutant domains were kept at RT for a week until full H/D exchange prior to PRE experiments. MTSL‐labeled SNARE complexes and Complexin‐1 (26–83) were prepared in the same buffer as mutant C_2_B domains, and both were concentrated to > 350 μm to minimize dilution of the sample upon addition of the complex. All the PRE experiments started with acquiring a ^1^H‐^15^ N TROSY‐HSQC of 33 μm Syt1‐ C_2_B mutant (C_2_B^RK‐EE^ or C_2_B^RKEE‐EEQQ^) alone in the presence of 1 mm EDTA. The complex formed by SNARE complex and complexin‐1 (26–83) at equimolar concentrations was formed prior to addition to Syt‐1 C_2_B domain mutant and supplemented with EDTA so that the final concentration of EDTA in the NMR sample was 1 mm. Complex of SNARE/complexin‐1 (26–83) or SNARE^Q^/complexin‐1 (26–83) (below referred to as SCCpx or SC^Q^Cpx) with MTSL label either on SNAP‐25 E27C or Synaptobrevin‐2 D44C was titrated to the mutant C_2_B domain and a ^1^H‐^15^ N TROSY‐HSQC was acquired at each titration step, resulting in 20, 50, 80 and 110 μm concentrations of SCCpx or SC^Q^Cpx. After acquiring spectra of the last titration step, the concentrations were 20 μm C_2_B and 110 μm SCCpx or SC^Q^Cpx (referred to as oxidized sample). The paramagnetic activity of MTSL was then removed by reduction with 1 mm ascorbic acid and 1 mm sodium dithionite (which reduces disulfide bonds) both added from 100 mm stocks. Sodium dithionite and ascorbic acid stocks were always prepared fresh before addition. The pH of the samples was always adjusted to 7.4 to minimize possible chemical shift changes due to alteration of the pH change. After reducing MTSL label, additional ^1^H‐^15^ N TROSY‐HSQC spectra were acquired (referred to as reduced sample).

PREs were calculated as the intensity ratio of the reduced and oxidized samples for each cross‐peak. Two ^1^H‐^15^ N TROSY‐HSQC spectra of C_2_B^RKEE‐RRQQ^ and SC/Cpx and C_2_B^RK‐EE^ and SC^Q^/Cpx were acquired for 3.5 h for both oxidized and reduced samples, and the mean PRE value plus standard derivation was plotted on the bar diagrams (Fig. 6) and the scatter plots (Fig. 7). Two spectra of C_2_B^RK‐EE^ and SC/Cpx were acquired for 3.5 h for both oxidized and reduced samples and added. In this condition, the PRE value calculated for the added spectra was plotted on the bar diagrams and the scatter plots. All the plots were prepared in GraphPad Prism (GrahPad Prism Software, San Diego, CA, USA). Mass spectrometry of oxidized samples confirmed that the SNARE complex was labeled with MTSL during NMR experiments prior to reduction. The dependence of PRE on the MTSL‐nucleus distance (Fig. 7) and the estimates of complex populations based on the observed PREs mentioned in the text were calculated with the equations described in [66] assuming a correlation time of 40 ns and a linewidth of 25 Hz (estimated from the cross‐peaks).

### Animals, primary hippocampal cultures, and lentivirus production

Pregnant Sprague‐ Dawley rats were housed individually until they gave birth to a litter. Postnatal day 2–3 Sprague–Dawley rats of either sex were used to prepare primary dissociated hippocampal cultures. The detailed description of animal care, preparation of primary dissociated hippocampal cultures, and packaging of knockdown and rescue expression constructs into lentiviruses to infect primary cultures are described in our previous work [67, 68]. All animal procedures were performed in accordance with the guide for the care and use of laboratory animals and were approved by the Institutional Animal Care and Use Committee at Vanderbilt University. The health status of the live animals was periodically checked and confirmed by the veterinary staff of animal facilities at the Vanderbilt University.

### Protein quantification in hippocampal cultures

To quantify protein levels in primary dissociated hippocampal cultures, western blotting was carried out. Briefly, protein samples were prepared from coverslips using Laemmli Buffer containing protease and phosphatase inhibitor cocktails (Roche) and beta‐mercaptoethanol. Samples were sonicated for 15 min and loaded on SDS/PAGE gels and transferred to nitrocellulose membranes. Membranes were incubated with primary antibodies at 4 °C overnight in the following dilutions: 1 : 10 000 anti‐GAPDH rabbit (14C10, Cell Signaling, Danvers, MA, USA) and 1 : 1000 anti‐synaptotagmin‐1 mouse (105‐011, Synaptic Systems, Goettingen, Germany). After incubation with fluorescent secondary antibodies (IRDye Secondary Antibodies, Licor Biosci, Lincoln, NE, USA), membranes were imaged using an Odyssey CLx imaging system (Licor). Band intensities were analyzed using imagej and normalized to loading controls.

### Immunofluorescence

Coverslips were fixed in 4% PFA/4% sucrose‐containing PBS solution for 20 min at room temperature and permeabilized using 0.2% Triton X‐containing PBS solution for 30 min at room temperature. Coverslips were washed and incubated in a blocking solution consisting of 1% bovine serum albumin and 2% goat serum in PBS for 2 h at room temperature. Coverslips were incubated with primary antibodies diluted in the blocking solution: 1 : 500 anti‐synapsin (106‐103, Synaptic Systems) and 1 : 500 anti‐synaptotagmin‐1 mouse (105‐011, Synaptic Systems) in a humid chamber overnight at 4 °C and with species‐appropriate Alexa Fluor secondary antibodies diluted as 1 : 500 in the blocking solution in a humid chamber for 90 min at room temperature. Coverslips were mounted on glass slides and imaged via a Zeiss LSM 710 (Zeiss, Oberkochen, Germany) with 63× objective at 1024 × 1024‐pixel resolution. The co‐localization of Synaptotagmin‐1 signal with synapsin signal was assayed with ImageJ (imagej.nih.gov) and expressed using Pearson's correlation coefficient.

## Results

The complexities of studying interactions between Syt1 and the SNARE complex are particularly well illustrated by NMR experiments that we performed years ago to build on studies showing that the Syt1 C_2_B domain or a fragment spanning the two Syt1 C_2_ domains (C_2_AB) could displace a complexin‐1 fragment spanning the accessory and central helices [Cpx1(26‐83)] from SNARE complexes anchored on supported lipid bilayers in the presence of Ca^2+^ [26, 55]. Mutagenesis showed that such displacement required the C_2_B domain polybasic region and acidic residues at the C‐terminus of SNAP‐25 (D186 and D193). To attempt to define the structural basis for the C_2_B domain‐SNARE interactions underlying these results, we used an NMR approach based on the measurement of paramagnetic relaxation enhancements (PREs) caused by MTSL spin labels. These experiments were performed in the absence of Ca^2+^, which induces the precipitation of Syt1‐SNARE complexes [54, 55]. Based on previous data [55, 61], we anticipated that C_2_AB‐SNARE complex binding is weak under these conditions, but we took advantage of the high sensitivity of the PRE‐based approach, as MTSL attached to a protein causes very strong relaxation effects on nuclei from another protein that come into proximity upon binding even when the bound population is very low [66, 69]. We attached MTSL to different positions of the C‐terminal half of the SNARE complex four‐helix bundle (Fig. 2A, Fig. S1A) and measured the PREs caused on ^1^H‐^13^C HMQC spectra of ^15^ N,^2^H–labeled Syt1 C_2_AB specifically ^1^H‐^13^C‐labeled at isoleucine, leucine and valine methyl groups (^15^ N‐^2^H–ILV‐^13^CH_3_–labeled), which exhibit high sensitivity even for large protein complexes [70].

Figure 2B illustrates the data obtained when MTSL was placed on residue 65 of SNAP‐25 in the SNARE complex and shows that many cross‐peaks of the C_2_B domain were strongly broadened, some beyond detection. Substantial PREs were also observed for many C_2_B domain cross‐peaks when MTSL was placed on residues 61 or 72 of synaptobrevin, or residue 239 of syntaxin‐1, whereas less extensive broadening was observed with MTSL on residue 187 or 197 of SNAP‐25 (Fig. 2C–H, Fig. S2). To interpret these data, it is important to consider that strong PREs (< 0.4) should be observed only for MTSL‐methyl distances below 19 Å under the conditions of our experiments, based on the correlation time expected for the SNARE‐C_2_AB complex [66] and assuming quantitative binding (see Materials and methods). Because the distance between residue 61 of synaptobrevin and residue 65 of SNAP‐25 in the SNARE complex is over 20 Å, and because MTSL attached to each one of these residues induced strong PREs on methyl groups located on the Ca^2+^‐binding loops, on the β‐sheet containing the polybasic region and on the other β‐sheet containing the primary interface, it is clear that the PRE data cannot be explained by a single binding mode between C_2_AB and the SNARE complex. It is also important to note that PREs below 0.2 can result when the MTSL probe comes within < 10 Å of a nucleus even if a complex is only 10% populated, or when the probe comes within < 5 Å even if the complex population is only 0.1%. Hence, the most likely explanation for the observed PREs is that there are multiple binding modes that are partially populated and alternatively involve the polybasic region, the primary interface, or the Ca^2+^‐binding loops. Because each one of these regions contains basic residues and the SNARE complex has abundant negatively charged patches, the interactions underlying the observed PREs are most likely nonspecific. However, we cannot rule out the possibility that one of the lowly populated binding modes is physiologically relevant.

Importantly, the C_2_B domain‐SNARE complex binding modes defined by NMR spectroscopy and X‐ray crystallography after these PRE experiments were performed [30, 31, 54] (Fig. 1B–D) are not consistent with our PRE data because the MTSL‐methyl distances predicted from these binding modes are generally too long to yield observable PREs, further emphasizing the multiplicity of ways by which Syt1 can bind to the SNARE complex. Note, however, that these binding modes revealed by the three structures are all compatible with complexin‐1 binding to the SNARE complex. These observations support the notion that the Syt1‐SNARE complex interactions that cause the displacement of Cpx1(26‐83) from membrane‐anchored SNARE complexes by C_2_AB involve the C‐terminal half of the SNARE complex four‐helix bundle [55], rather than the SNARE complex surfaces that mediate Syt1 binding in the three structures. At least some of the binding modes underlying the PREs summarized in Fig. 2 and Fig. S2 are most likely incompatible with the binding of Cpx1 (28–83) to the SNARE complex and underlie Cpx1(26‐83) displacement, as expected because the binding of the C_2_B domain around the area where we placed the MTSL labels would cause steric clashes with the Cpx1(26‐83) accessory helix.

### Re‐examination of the tripartite interface by ITC

^1^H‐^15^ N TROSY‐HSQC spectra of the Syt1 C_2_B domain acquired in the presence of increasing concentrations of SNARE complex bound to Cpx1(26‐83) revealed shifts in multiple cross‐peaks corresponding to residues from the polybasic region or the primary interface, showing that these two surfaces of the C_2_B domain are responsible for binding to the Cpx1(26‐83)‐SNARE complex [33]. However, there was no evidence for binding to the tripartite interface in these experiments, and binding was completely abolished by a quadruple R322E/K325E/R398Q/R399Q mutation designed to abrogate interactions involving both the polybasic and primary interfaces [33]. These findings led us to re‐examine whether binding through the tripartite interface could be observed by the ITC approach described in [31]. These experiments were performed with the same protein fragments used in that study [31], namely: (a) Syt1 C_2_B domain bearing seven mutations (K326A/K327A/R281A/E295A/Y338W/R398A/R399A) designed to disrupt binding to the SNARE complex through the polybasic and primary interfaces (referred to as C_2_B^KA‐Q^); (b) SNARE complex bearing five mutations in SNAP‐25 (K40A/D51A/E52A/E55A/D166A) also designed to disrupt these interactions (referred to as SNARE^Q^); (c) a complexin‐1 fragment spanning residues 48–73, which include the central helix [Cpx1(48‐73)]; and (d) Syt1 C_2_B domain with the same seven mutations plus an additional L387Q/L394Q mutation (LLQQ) designed to disrupt the tripartite interface (referred to as C_2_B^KA‐Q‐LLQQ^) (Fig. S1B). The proteins were purified by the same protocols as in [31] except that purification of the Syt1 C_2_B constructs with KA‐Q mutations included additional steps such as protamine sulfate precipitation, nuclease treatment, and ion exchange chromatography (see Materials and methods), which proved crucial to remove polyacidic contaminants that have a high tendency to bind to the C_2_B domain [61, 71]. Moreover, the C_2_B domain mutants were uniformly ^15^ N‐labeled such that the same samples used in the ITC experiments could be examined by NMR spectroscopy. ^1^H‐^15^ N TROSY‐HSQC spectra of C_2_B^KA‐Q^ and C_2_B^KA‐Q‐LLQQ^ acquired in the absence and presence of Ca^2+^ confirmed that both proteins are properly folded and bind to Ca^2+^ (Figs S3 and S4).

It is worth noting that the ion exchange step was not used for the purification of the C_2_B constructs with KA‐Q mutations in ref. [31] because these mutants appeared in the flow‐through under the conditions that were used at that time. By contrast, the buffers used to purify the C_2_B constructs with KA‐Q mutations for the experiments described here contained 20 mm Ca^2+^ and the proteins bound to the ion exchange resin under these conditions. We note that all other Syt1 C_2_B constructs studied in ref. [31], including WT C_2_B and the C_2_B^KA^ mutant, included an ion exchange step. Hence, the absence of an ion exchange chromatography step for purification applies only to the ITC studies of the C2B mutants with KA‐Q mutations, which provided the key evidence for binding through the tripartite interface in solution in ref. [31]. In these studies, an endothermic ITC signal was observed when C_2_B^KA‐Q^ was titrated onto the SNARE^Q^‐Cpx1(48‐73) complex that was not observed in the absence of Cpx1(48‐73) and was strongly decreased by the LLQQ mutation [31]. In addition to suggesting that the endothermic ITC signal arose from the binding of C_2_B^KA‐Q^ to the SNARE^Q^‐Cpx1(48‐73) complex through the tripartite interface, these results indicated that such binding required Cxp1(48‐73).

However, the ITC curves that we observed upon titration of C_2_B^KA‐Q^ on SNARE^Q^ either in the absence or presence of Cpx1(48‐73) using the same conditions as in [31] exhibited a biphasic behavior with a slightly exothermic signal (Fig. 3A,B). Analogous data were obtained when C_2_B^KA‐Q‐LLQQ^ was titrated onto SNARE^Q^ (Fig. 3C). These results were reproduced in multiple independent experiments performed with different protein preparations in the Rizo and the Brunger laboratories (Fig. S5). Additional titrations monitored by ITC confirmed that, as expected, Cpx1(48‐73) binds to SNARE^Q^ but not to C_2_B^KA‐Q^ (Fig. S6).

Comparison of the ^1^H‐^15^ N TROSY‐HSQC spectra of the ITC samples containing C_2_B^KA‐Q^ and SNARE^Q^ with spectra acquired for isolated C_2_B^KA‐Q^ revealed that the presence of SNARE^Q^ induced similar, small cross‐peak perturbations regardless of the presence or absence of Cpx1(48‐73) (Fig. 4A,B). Since the multiple mutations in C_2_B^KA‐Q^ caused shifts in many cross‐peaks compared with the WT C_2_B domain spectrum (Fig. S3A), we assigned the backbone resonances of C_2_B^KA‐Q^ using triple resonance experiments to facilitate analysis of changes in the ^1^H‐^15^ N TROSY‐HSQC spectra (Fig. S7). The cross‐peaks that were perturbed by the binding of SNARE^Q^ to C_2_B^KA‐Q^ correspond mostly to residues in the β‐sheet that forms the primary interface (Fig. 4C). These results likely arise because the KA‐Q mutation includes a Y338W substitution that enhances the binding of the C_2_B domain to the SNARE complex [33], possibly by shifting the conformation of the primary interface. It is noteworthy that cross‐peak perturbations were also observed for residues at the N‐terminus of helix HA, which forms part of the tripartite interface (Fig. 4C). However, such perturbations can be attributed to the proximity of these residues to the residues of the β‐sheet that are most markedly affected by SNARE^Q^ binding to C_2_B^KA‐Q^ through Y338W. The absence of perturbations in the rest of the cross‐peaks corresponding to HA shows that the tripartite interface cannot be detected in these experiments. Analysis of cross‐peak perturbations caused by the SNARE^Q^‐Cpx1(48‐73) complex on C_2_B^KA‐Q‐LLQQ^ was hindered by the additional shifts caused by the LLQQ mutation but, overall, the perturbations (Fig. 4D) were comparable to those observed for C_2_B^KA‐Q^ (Fig. 4A). These results are consistent with the observation that the LLQQ mutation does not substantially affect the ITC signal (Fig. 3) and support the conclusion that the small heat observed in the ITC experiments does not arise from binding to the tripartite interface.

### Analysis of potential tripartite Syt1‐SNARE‐complexin‐1 complexes using PREs

Our ITC and NMR experiments using Syt1 C_2_B^KA‐Q^ purified by ion exchange chromatography did not detect an interaction of Syt1 C_2_B^KA‐Q^ with SNARE^Q^ through the tripartite interface in the absence or presence of Cpx1(48‐73). These results suggest that binding of the Syt1 C_2_B domain to the SNARE complex through the tripartite interface is very weak even when the SNARE complex is bound to complexin‐1, consistent with previous experiments in which no binding of the Cpx1(26‐83)‐SNARE complex to the quadruple R322E/K325E/R398Q/R399Q Syt1 C_2_B domain mutant (below referred to as C_2_B^RKRR‐EEQQ^) was detected by NMR spectroscopy [33]. Since measurement of MTSL‐induced PREs provides a powerful tool to probe the presence of weak protein complexes in solution even when they are not detected by perturbations in ^1^H‐^15^ N HSQC spectra (e.g., [72]), we used this approach to examine whether we could detect binding of the Cpx1(26‐83)‐SNARE complex to C_2_B^RKRR‐EEQQ^ through the tripartite interface. In these experiments, we used ^1^H‐^15^ N TROSY‐HSQC experiments rather than methyl ^1^H‐^13^C HMQC spectra because they contain one cross‐peak for each nonproline residue in a protein and hence they provide more probes to thoroughly monitor binding involving any surface of the protein. To optimize the probability of observing PREs arising from binding through the tripartite interface, we placed MTSL on two distinct positions of the SNARE complex that are expected to be close to the tripartite interface with the C_2_B domain but without interfering with binding through this interface: (a) residue 44 of synaptobrevin, which is expected to be close to helix HA of the C_2_B domain on one side of the tripartite interface (Fig. 1 E); and (b) residue 27 of SNAP‐25, which is close to a short helix of the C_2_B domain on the opposite side of the tripartite interface (Fig. 1F; Fig. S1C). Before adding them to C_2_B^RKRR‐EEQQ^, the MTSL‐tagged SNARE complexes were mixed with Cpx1(26‐83). Below we refer to the assemblies of the MTSL‐tagged SNARE complexes bound to Cpx1(26‐83) as SCCpx D44C and SCCpx E27C, respectively.

PREs were measured from ^1^H‐^15^ N TROSY‐HSQC spectra acquired on samples containing 20 μm C_2_B^RKRR‐EEQQ^ that was ^15^ N,^2^H–labeled, in the presence of 110 μm MTSL‐labeled SCCpx D44C or SCCpx E27C (Figs 5A,B and 6A,B). Most PREs were in the 0.8–1.2 range, and those in the low end of this range were not concentrated in any particular region of the C_2_B domain mutant, indicating that they arise at least in part from natural variability in cross‐peak intensities. Note that a PRE of 0.8 can arise when the MTSL probe comes within 5 Å of an NH group in just 0.01% of the molecules. Hence, it is very difficult to draw any conclusions about the binding modes underlying such PREs unless they are concentrated in a particular region of a protein. SCCpx D44C induced PREs below 0.8 for only the backbone NH cross‐peaks from residues 305 and 330, which are located in the Ca^2+^‐binding loops, and for the cross‐peak from the W404 side chain (Fig. 6A, Fig. S8A). In the presence of SCCpx E27C, PREs below 0.8 were observed again for the W404 side chain NH and for the backbone NH groups corresponding to residues 295 and 342, which are located in the β‐sheet that forms the primary interface (Fig. 6B, Fig. S8B). The W404 side chain is surface exposed and may occasionally come into close contact with the MTSL probe (see [66]), while the paucity of backbone NH groups exhibiting PREs below 0.8 suggests that these PREs may also arise from sporadic nonspecific encounters rather than from specific binding modes. Hence, these PRE experiments did not yield any evidence for binding of the C_2_B^RKRR‐EEQQ^ mutant to the SNARE complex through the tripartite interface in solution.

This conclusion is further supported by plots of the observed PREs versus the approximate distance between the corresponding NH and the MTSL group predicted if the C_2_B domain quadruple mutant was bound to SCCpx D44C or SCCpx E27C via the primary or tripartite interfaces, comparing them with the theoretical dependence of PREs on the MTSL‐NH distance [66] (Fig. 7A,D,G,J). The plots involving distances predicted with the primary interface (Fig. 7A,D) are inconclusive with regard to binding through this interface because the positions of the MTSL probes were not designed to monitor this binding mode and they would be more than 22 Å away from all NH groups. Hence, the fact that most PREs are above 0.8 does not provide useful information about binding. Conversely, the plots involving distances predicted from the tripartite interface (Fig. 7G,J) show that strong PREs (< 0.4) would be expected for multiple NH groups predicted to be at distances of less than 19 Å from the MTSL probe if there was binding through this interface, but no such strong PREs were observed. The absence of a strong SCCpx D44C‐induced PRE even for the NH group of residue 384, which is expected to be less than 11 Å away from the MTSL group in the tripartite complex (predicted PRE 3 × 10^−6^), shows that there is no substantial binding through the tripartite interface under the conditions of these experiments.

Since in MD simulations of the tripartite interface we observed that the R398 side chain sometimes interacts with the SNAREs in this binding mode [73], it is plausible that the failure to detect binding of C_2_B^RKRR‐EEQQ^ to the SNARE complex through the tripartite interface might arise because this mutant bears an R398Q substitution. Hence, we examined whether binding through the tripartite interface might be observable for the R322E/K325E C_2_B mutant, in which the polybasic interface is disrupted but the primary and tripartite interfaces are unaltered (below referred to as C_2_B^RK‐EE^). Thus, this mutant still binds to the SNARE complex through the primary interface [33], but we reasoned that binding to the tripartite interface could still be observable using a large excess of SCCpx D44C or SCCpx E27C since the two binding modes are compatible with each other. In the presence of 110 μm SCCpx D44C or SCCpx E27C, we observed a considerable number of moderate PREs below 0.8 and a strong PRE with the W404 side chain NH induced by SCCpx E27C (Figs 5C,D and 6C,D). However, these PREs involved residues from different regions of C_2_B^RK‐EE^ (Fig. 6C,D, Fig. S8C,D). We note again that binding to SCCpx D44C or SCCpx E27C through the primary interface is not expected to cause strong PREs because the probes should be more than 22 Å from all NH groups. The NH groups predicted to be closest to the MTSL probe of SCCpx D44C and SCCpx E27C in the primary complex are those of Y338 and N298, respectively, and the corresponding observed PREs were slightly smaller than 0.8, consistent with the expected MTSL‐NH distances (Figs 6C,D and 7B,E). However, other PREs below 0.8 are generally not consistent with the distances predicted from binding through the primary interface (Fig. 7B,E) and therefore are most likely to arise from other binding modes. Since the NH chemical shift changes induced by the Cpx1(26‐83)‐SNARE complex on the C_2_B^RK‐EE^ mutant are concentrated in the primary interface [33], the populations of these additional binding modes that yield PREs below 0.8 should be small.

Plots of the PREs induced by SCCpx D44C and SCCpx E27C on the C_2_B^RK‐EE^ mutant NH cross‐peaks versus the distances to the MTSL probe predicted from the tripartite complex showed that none of the strong PREs expected for this binding mode were observed (Fig. 7H,K). The NH group of K354, which is predicted to be closest to the MTSL group of SCCpx E27C (at < 10.6 Å) did exhibit a PRE below 0.8, but the PRE value (0.77) was far from that predicted from the tripartite complex (PRE 2 × 10^−7^). Moreover, the PREs observed for other nearby NH groups that are expected to be within 11–12 Å of the MTSL group of SCCpx E27C (those of residues 350, 351, and 353) were 0.89, 1.10, and 1.07, respectively. Assuming that the PREs observed for the NH groups of residues 350 and 354 actually arose from binding through the tripartite interface and that their distances to the MTSL in the tripartite complex are 11.6 and 10.6 Å, respectively, the populations of the tripartite complex that can be calculated from these PREs are 0.7% and 0.9%, which correspond to *K*_D_s of 16 and 12 mm, respectively. There is an uncertainty in these affinity calculations given the assumptions made (see Materials and methods). However, the overall PRE data obtained with the C_2_B^RK‐EE^ mutant does provide strong evidence that, if the tripartite complex forms in solution, it has a very low affinity, with a *K*_D_ in the low millimolar range or higher. This is corroborated by molecular dynamics simulations of both the primary and tripartite interfaces [73], which suggest lower stability of the tripartite interface on the 1‐microsecond timescale and are consistent with this estimate.

To address the possibility that binding to the tripartite interface in the PRE experiments with the C_2_B^RK‐EE^ mutant might have been hindered by the interaction involving the primary interface, we performed additional PRE measurements with this mutant and an assembly of Cpx1(26‐83) with SNARE complex that contained the quintuple K40A/D51A/E52A/E55A/D166A mutation designed to disrupt the primary interface (SNARE^Q^) and was again tagged with MTSL at residue 44 of synaptobrevin or residue 27 of SNAP‐25 (referred to as SC^Q^Cpx D44C and SC^Q^Cpx E27C, respectively). The PRE patterns observed in these experiments (Figs 5E,F, 6E,F and 7C,F,I,L and S8E,F) exhibited some similarities and differences with respect to those observed for C_2_B^RK‐EE^ in the presence of SCCpx D44C or SCCpx E27C, and SC^Q^Cpx E27C induced markedly stronger PREs than SCCpx E27C. These data suggest that some similar and some distinct binding modes are visited in the samples of the C_2_B^RK‐EE^ mutant containing SCCpx or SC^Q^Cpx. Although the PRE induced by SC^Q^Cpx D44C on the G384 NH (PRE 0.59) might suggest that there is a small population of tripartite complex because this NH is expected to be close to the MTSL probe (at 10.4 Å) in this complex, the PREs observed for nearby NH groups of the HA helix did not confirm this conclusion. It is plausible that the PRE observed for G384 NH arises because of its proximity to the β‐strand containing W404, which also exhibits PREs below 0.8. Note also that the PRE induced by SC^Q^Cpx E27C on G384 NH is similar (0.56) even though this NH group is expected to be at 23 Å from the MTSL probe in the tripartite complex with SC^Q^Cpx E27C.

Overall, the PRE data did not provide evidence for the existence of substantial populations of Syt1 C_2_B domain‐SNARE‐complexin‐1 complexes involving the tripartite interface, although they do not rule out the possibility that these complexes are formed with a very low affinity (*K*_D_ >1 mm). The PRE data do corroborate the notion that there are several binding modes between the Syt1 C_2_B domain and the SNARE complex that are distinct from the primary complex, but some of these complexes involve the β‐sheet involved in the primary interface, as multiple PREs below 0.8 were observed in this region in the different data sets. Most of these binding modes likely involve the arginine‐rich region containing R398 and R399, as fewer PREs below 0.8 were observed in the experiments performed with the quadruple C_2_B^RKRR‐EEQQ^ mutant than in those involving the double C_2_B^RK‐EE^ mutant. Since the experiment with the latter mutant and SC^Q^Cpx E27C exhibited somewhat stronger PREs than those performed with SCCpx E27C, we investigated whether the additional binding modes that are visited with the SNARE^Q^ complex depend on the presence of complexin‐1 using ITC. Interestingly, titrations of SNARE^Q^ with the C_2_B^RK‐EE^ mutant in the absence of Cpx1(48‐73) yielded an endothermic ITC signal that, although weak, was consistently stronger than that observed in the presence of Cpx1(48‐73) (Fig. 8, Fig. S9). These results suggest that some of the C_2_B^RK‐EE^‐ SNARE^Q^ binding modes underlying the endothermic ITC signal compete with the binding of Cpx1(48‐73) to SNARE^Q^ and might be related to the binding modes observed in our PRE experiments monitored by ^1^H‐^13^C HMQC spectra (Fig. 2). These results once again emphasize the difficulties involved in characterizing Syt1‐SNARE complex interactions.

### The Syt1 LLQQ mutant is properly trafficked to the synapse

The physiological relevance of the tripartite interface was in principle supported by experiments performed with two Syt1 mutants bearing residue substitutions at the tripartite interface: the LLQQ mutant mentioned above, and a T383Q/G384Q mutant referred to as TGQQ [31]. The LLQQ mutant but not the TGQQ disrupted neurotransmitter release, and these results correlated with the ITC data that suggested that the LLQQ mutation disrupted the binding of C_2_B^KA‐Q^ to SNARE^Q^‐Cpx1(48‐73) while the TGQQ mutation did not [31]. However, the results presented here show that we cannot detect the binding of C_2_B^KA‐Q^ to SNARE^Q^‐Cpx1(48‐73) or to SNARE^Q^ through the tripartite interface by ITC or NMR spectroscopy, and the interaction between C_2_B^KA‐Q^ and SNARE^Q^‐Cpx1(48‐73) through the primary interface that we did observe was not affected by the LLQQ mutation (Figs 3B,C and 4B,D). Moreover, some evidence suggested that the LLQQ mutation destabilizes the C_2_B domain [33]. These observations raise the question as to whether the disruption of neurotransmitter release caused by the LLQQ mutation might have arisen from Syt1 mislocalization. To address this question, we used primary dissociated hippocampal cultures infected with either sham lentivirus or lentivirus carrying siRNA to knockdown Syt1 and to express mCherry [68]. Syt1 knockdown efficacy was more than > 90% (Fig. S10A). This conclusion was also confirmed visually from the loss of Syt1 signal observed with immunocytochemistry, together with the emergence of mCherry introduced by the knockdown construct. We then expressed WT Syt1 or Syt1 TGQQ and LLQQ mutants on the Syt1 knockdown background. To confirm that the Syt1 mutants are not only expressed but also trafficked to the synapse, where they are expected to function, we co‐stained Syt1 with synapsin, a synaptic marker, and performed co‐localization analyses. We observed that exogenously introduced WT Syt1, TGQQ Syt1, and LLQQ Syt1 co‐localized with synapsin to similar extents as the endogenous Syt1 in control cultures (Fig. S10B). These results suggest that the physiological effects caused by the LLQQ mutation arise from a true loss of function rather than from a trafficking defect.

## Discussion

Elucidating how Syt1 interacts with the SNARE complex is crucial to understand how Ca^2+^ sensing is coupled to membrane fusion during neurotransmitter release, but it is clear that diverse binding modes between Syt1 and the SNAREs exist *in vitro*. Such diversity arises in part from the promiscuity of Syt1 and the SNAREs, but it is also plausible that distinct Syt1‐SNARE complex binding modes occur during the steps that lead from synaptic vesicle tethering to priming and finally Ca^2+^‐triggered fusion. Moreover, more than one binding mode might occur simultaneously. Thus, a major challenge is to characterize each interaction mode and to distinguish which of the interactions is physiologically relevant. X‐ray crystallography provided crucial insights into this area by revealing the Syt1 C_2_B domain‐SNARE complex binding mode mediated by the primary interface [30], the physiological relevance of which is supported by overwhelming evidence. In addition, X‐ray crystallography revealed another C_2_B domain‐SNARE complex binding mode that is mediated by the tripartite interface and also involves complexin‐1 [31]. While ITC data performed with Syt1 C_2_B constructs bearing the KA‐Q mutations supported the presence of this binding mode in solution in this study, subsequent NMR experiments did not detect binding through the tripartite interface [33]. Here we have tried to clarify this apparent contradiction and show that the same ITC approach does not provide evidence for the presence of the tripartite binding mode in solution when the purification of the Syt1 C_2_B constructs with KA‐Q mutations included ion exchange chromatography. Consistent with the new ITC results presented here, we could not detect binding through the tripartite interface even through a PRE‐based NMR approach that has a high sensitivity for detection complexes of low affinity. However, we find that Syt1 bearing the LLQQ mutation at the tripartite interface is properly localized to synapses, suggesting that the disruption of neurotransmitter release caused by this mutation does not arise from mislocalization but rather from physiological loss of function of the LLQQ mutant at the synapse. Overall, these results show that further research will be required to determine whether the tripartite interface is physiologically relevant and emphasize the need to develop novel tools to study very weak Syt1‐SNARE interactions that may be functionally relevant, ideally between two membranes.

The stoichiometry of Syt1 and the complexin‐1‐SNARE complex in the crystals that revealed the tripartite interface was 1 : 1, and Syt1 also interacted with the SNARE complex through the primary interface in these crystals [31]. Since the critical functional importance of the primary interface is well established, the tripartite interface might in principle constitute a crystallization artifact. However, the tripartite interface is very similar in two distinct crystal forms, it involves a relatively large surface area (990 Å^2^) and has well‐defined densities for most side chains, which is not typical of interactions that merely constitute crystal contacts. Moreover, the fact that the tripartite interface involves interactions of the Syt1 C_2_B domain with both the SNARE complex and complexin‐1 provides attractive explanations for the cooperation between Syt1 and complexin‐1 in inhibiting neurotransmitter release and for the finding that complexins are required for the dominant negative effect observed upon overexpression of Syt1 bearing mutations that abolish Ca^2+^ binding to the C_2_B domain (referred to as Syt1^DA^) [31]. Note, however, that these findings can also be explained by the interaction of Syt1 with the SNARE complex through the primary interface, as simultaneous binding of the C_2_B domain to the SNARE complex through this interface and to the plasma membrane through the polybasic region dictates that the accessory helix of complexin (bound to the SNARE complex) has steric clashes with the vesicles that are expected to hinder final C‐terminal assembly of the SNARE four‐helix bundle [33, 34].

The most compelling evidence supporting the functional relevance of the tripartite interface was provided by the strong physiological effects of the LLQQ mutation at the tripartite interface, which disrupted Ca^2+^‐evoked neurotransmitter release and the dominant negative effects of Syt1^DA^. Here, we show that the Syt1 LLQQ mutant is properly localized to the synapse (Fig. S10), and therefore that these phenotypes are unlikely to arise from mislocalization. The TGQQ mutation, which also replaces residues at the tripartite interface, did not disrupt evoked neurotransmitter release or the dominant negative effects of Syt1^DA^. These phenotypes correlated with the results of the ITC assays that appeared to show disruption of Syt1 C_2_B^KA‐Q^ binding to the SNARE^Q^‐Cpx1(48‐73) by the LLQQ mutation but not the TGQQ mutation. However, we could not reproduce these results when C_2_B^KA‐Q^ was purified by ion exchange chromatography (Fig. 3, Fig. S5), which is a critical step to eliminate polyacidic contaminants that have a high tendency to bind to the C_2_B domain [71]. Moreover, we did not detect binding of C_2_B^KA‐Q^ to SNARE^Q^ or SNARE^Q^‐Cpx1(48‐73) through the tripartite interface in the ITC samples by NMR spectroscopy (Fig. 4). It is also important to note that the observation that Cpx1(48‐73) was required to observe binding of C_2_B^KA‐Q^ to SNARE^Q^ in the ITC assays supported the notion that Syt1‐SNARE complex binding through the tripartite interface underlies the cooperation of Syt1 and complexins in inhibiting neurotransmitter release, as well as the requirement of complexins for the dominant negative effects of Syt1^DA^ [31]. However, our ITC and NMR results were not substantially affected by Cpx1(48‐73) or by the LLQQ mutation (Figs 3 and 4, Fig. S5).

ln summary, neurotransmitter release is strongly disrupted by one mutation in the tripartite interface, LLQQ, but not by another, TGQQ, and currently, there is no reliable assay in solution to monitor the binding of Syt1 to the SNARE complex through the tripartite interface. Hence it is not possible with currently available assays to test whether the effects of mutations on binding through this interface correlate with physiological effects in neurons. ITC is without a doubt a very powerful tool to analyze the thermodynamics of tight protein interactions, but its application to interactions that are weak or yield small heats, or when multiple interactions are present, is more problematic because of potential interference from unexpected impurities or from sequences with a propensity to nonspecific binding such as His_6_‐tags [74]. Our results provide a vivid illustration of this problem. For this reason, we turned to NMR methods that are highly sensitive for the detection of weak protein interactions. However, we were not able to detect the binding of WT or mutant Syt1 C_2_B domain to the SNARE‐Cpx1(26‐83) complex or the SNARE^Q^‐Cpx1(48‐73) complex via the tripartite interface through analysis of ^1^H‐^15^ N TROSY‐HSQC perturbations or PREs even at SNARE complex concentrations on the 100 μm range (Figs 4, 5, 6, 7) and ref. [33]. As mentioned above, the absence of conclusive evidence for such binding in our PRE experiments indicates that the tripartite interface interaction is very weak, with a *K*_D_ in the low millimolar range or higher. However, it is plausible that this interaction might be enhanced *in vivo* by co‐localization of the proteins in the small volume around a docked synaptic vesicle and/or by cooperativity with other interactions, for instance, interactions of Syt1 with the membranes.

Our PRE data clearly show that there are multiple interaction modes between Syt1 and the SNARE complex beyond those involving the primary and tripartite interfaces (Figs 2 and 4, 5, 6, 7, Figs S2 and S8). Unfortunately, because of the multiplicity of binding modes and the high sensitivity of PRE detection even for complexes that are sparely populated, the PRE data do not allow an accurate definition of the binding modes. A dominant, dynamic binding mode involving interactions of the Syt1 C_2_B domain polybasic region with a polyacidic region in the middle of the SNARE complex was elucidated by NMR spectroscopy using lanthanide‐induced pseudocontact shifts, and its physiological relevance was supported by the correlation between the effects of four mutations in basic residues on Syt1‐SNARE complex binding *in vitro* with those caused on neurotransmitter release in neurons [54]. However, it now seems clear that the physiological effects observed in this study can also be explained because of the disruption of Ca^2+^‐dependent interactions of the Syt1 C_2_B domain polybasic region with PIP_2_‐containing membranes such as the plasma membrane, which are much tighter than the C_2_B‐SNARE complex interactions [33]. The Syt1 C_2_B domain polybasic region also interacts with the C‐terminal half of the SNARE complex and these interactions underlie the finding that the Syt1 C_2_AB fragment can displace Cpx1(26‐83) from membrane‐anchored SNARE complexes in the presence of Ca^2+^ [26, 55]. These interactions likely underlie at least to some extent the PREs that we observed when we placed MTSL on different positions of the C‐terminal half of the SNARE complex (Fig. 2, Fig. S2), and led to the attractive idea that, upon Ca^2+^ influx, binding of Syt1 to the C‐terminus of the SNARE complex releases the inhibition of neurotransmitter release caused by the complexin accessory helix [55]. However, there is no high‐resolution structural information on these interactions and it is unclear whether they are compatible with tight Ca^2+^‐dependent binding of Syt1 to PIP_2_‐containing membranes. Moreover, there is evidence that Syt1 C_2_AB does not bind to SNARE complexes anchored on PIP_2_‐containing membranes in the presence of Ca^2+^, ATP, and physiological salt concentrations [33, 75].

The observation that the SNARE complex bound to Cpx1(26‐83) causes no marked perturbations on the ^1^H‐^15^ N TROSY‐HSQC spectrum of C_2_B^RKRR‐EEQQ^ mutant [33] and that SCCpx with an MTSL paramagnetic tag attached to D44C or E27C caused almost no PREs on this mutant (Figs 5A,B and 6A,B) showed that binding of the C_2_B domain to the SNARE complex is practically abolished by this quadruple mutation that impairs binding through the polybasic region and the arginine‐rich region of the primary interface. Hence, C_2_B domain‐SNARE complex binding is largely governed by ionic interactions involving these two regions. It is noteworthy that SC^Q^Cpx D44C and SC^Q^Cpx E27C still caused sizeable PREs on the ^1^H‐^15^ N TROSY‐HSQC spectrum of C_2_B^RK‐EE^ (Figs 5E,F and 6E,F) and that we observed some endothermic signal in the ITC experiments performed with the C_2_B^RK‐EE^ and SNARE^Q^ (Fig. 8, Fig. S9), even though the RK‐EE mutation is expected to disrupt interactions involving the polybasic region and the quintuple mutation in SNARE^Q^ is expected to abrogate binding through the primary interface. These findings show that there are additional binding modes involving the middle of the SNARE complex that are mediated at least in part by the arginine‐rich region, illustrating again the difficulties associated with characterizing Syt1‐SNARE complex interactions.

In summary, it is well established that binding of Syt1 to the SNARE complex through the primary interface plays a key function in neurotransmitter release, but further research will be necessary to determine whether the tripartite interface is physiologically relevant and whether other Syt1‐SNARE complex interactions that have not been well characterized might also play important roles. The diversity of such interactions that can be detected in solution arises in part because of the absence of phospholipids, the natural targets of the promiscuous polybasic region. Thus, further progress in this field will likely require the use of trans‐SNARE complexes bridging two membranes so that the configuration existing in primed vesicles can be mimicked as closely as possible. Such studies are currently feasible, as methods to prepare stable trans‐SNARE complexes bridging reconstituted proteoliposomes have been described (e.g., [13, 76, 77]), and reconstitution approaches that recapitulate key aspects of Ca^2+^‐triggered synaptic vesicle fusion with multiple components are available [2, 3, 4, 5]. Cryo‐electron microscopy and cryo‐electron tomography constitute powerful tools to study synaptic proteins bridging membranes [23, 57, 76, 77, 78, 79, 80], although the small size of Syt1, the SNAREs, and complexins hinder the application of these techniques to visualize their complexes with sufficient resolution at the moment. MD simulations provide an alternative means to examine the effects of mutations on Syt1‐SNARE complex *in silico* [73] and compare them with the physiological consequences of the same mutations. It seems likely that, with a combination of these and other approaches, a clear picture of how Syt1‐SNARE interactions control neurotransmitter release will emerge in a not‐too‐distant future.

## Conflict of interest

The authors declare no conflict of interest.

## Author contributions

KJ, QZ, ETK, ATB, and JR conceptualized and supervised the study, and designed the experiments. KJ, LE, RAP, BA, and KDB performed the experiments. KJ, QZ, ATB, and JR wrote the manuscript, and LE, RAP, BA, KDB, and ETK revised the manuscript.

## Supporting information


**Fig. S1.** Summary of mutants of the various presynaptic proteins used in this study.
**Fig. S2.** PRE analysis of Syt1 C_2_AB binding to the C‐terminal half of the SNARE complex.
**Fig. S3.** The C_2_B^KA‐Q^ mutant is properly folded and binds Ca^2+^.
**Fig. S4.** The C_2_B^KA‐Q‐LLQQ^ mutant is properly folded and binds Ca^2+^.
**Fig. S5.** ITC analysis of interactions between C_2_B^KA‐Q^ and SNARE^Q^.
**Fig. S6.** ITC analysis of interactions of Cpx1(48‐73) with SNARE^Q^ and C_2_B^KA‐Q^.
**Fig. S7.** Cross‐peaks assignments of the ^1^H‐^15^ N HSQC spectrum of C_2_B^KA‐Q^.
**Fig. S8.** Analysis of potential tripartite Syt1‐SNARE‐complexin‐1 complexes using PREs.
**Fig. S9.** ITC analysis of interactions between C_2_B^RK‐EE^ and SNARE^Q^.
**Fig. S10.** Analysis of the localization of the Syt1 LLQQ and TGQQ mutants.

## Data Availability

The source data that support the findings of this study are available from the corresponding author (jose.rizo-rey@utsouthwestern.edu) upon reasonable request.
