# Supplementary material for: Analysis of tripartite Synaptotagmin‐1‐SNARE‐complexin‐1 complexes in solution
Source: FEBS Open Bio. 2022 Nov 16;13(1):26–50. doi: 10.1002/2211-5463.13503 (PMC9811660; doi:10.1002/2211-5463.13503)
Supplement: Supplementary file 1 — Fig. S1. Summary of mutants of the various presynaptic proteins used in this study. Fig. S2. PRE analysis of Syt1 C2AB binding to the C‐terminal half of the SNARE complex. Fig. S3. The C2BKA‐Q mutant is properly folded and binds Ca2+. Fig. S4. The C2BKA‐Q‐LLQQ mutant is properly folded and binds Ca2+. Fig. S5. ITC analysis of interactions between C2BKA‐Q and SNAREQ. Fig. S6. ITC analysis of interactions of Cpx1(48‐73) with SNAREQ and C2BKA‐Q. Fig. S7. Cross‐peaks assignments of the 1H‐15 N HSQC spectrum of C2BKA‐Q. Fig. S8. Analysis of potential tripartite Syt1‐SNARE‐complexin‐1 complexes using PREs. Fig. S9. ITC analysis of interactions between C2BRK‐EE and SNAREQ. Fig. S10. Analysis of the localization of the Syt1 LLQQ and TGQQ mutants. [file FEB4-13-26-s001.pdf]

Supplementary Figures for:

Analysis of tripartite Synaptotagmin-1-SNARE-complexin-1 complexes in solution

Klaudia Jaczynska[1,2,3], Luis Esquivies[4,5,6,7,8], Richard A. Pfuetzner[4,5,6,7,8], Baris Alten[9,10,12], Kyle D. Brewer[1,2,3,13], Qiangjun Zhou[10,11], Ege T. Kavalali[9,10], Axel T. Brunger[4,5,6,7,8] and Josep Rizo[1,2,3]

[1]Department of Biophysics, University of Texas Southwestern Medical Center, Dallas, Texas 75390

[2]Department of Biochemistry, University of Texas Southwestern Medical Center, Dallas, Texas 75390

[3]Department of Pharmacology, University of Texas Southwestern Medical Center, Dallas, Texas 75390

[4]Department of Molecular and Cellular Physiology, Stanford University, Stanford, CA 94305

[5]Department of Neurology and Neurological Sciences, Stanford University, Stanford, CA 94305

[6]Department of Structural Biology, Stanford University, Stanford, CA 94305

[7]Department of Photon Science, Stanford University, Stanford, CA 94305

[8]Howard Hughes Medical Institute, Stanford University, Stanford, CA 94305

[9]Department of Pharmacology, Vanderbilt University, Nashville, TN, 37240-7933, USA

[10]Vanderbilt Brain Institute, Vanderbilt University, Nashville, TN, 37240-7933, USA

[11]Department of Cell and Developmental Biology, Vanderbilt University, Nashville, TN, 37240-7933, USA

[12]Current address: Massachusetts General Hospital, Department of Neurology, Boston, MA, 02114; Brigham and Women's Hospital, Department of Neurology, Boston, MA, 02115; Harvard Medical School, Boston, MA, 02115

[13]Current address: ETTA Biotechnology, Palo Alto, CA 94304

Supplementary Figures

Figure S1. Summary of mutants of the various presynaptic proteins used in this study. (A) SNAP-25, synaptobrevin and syntaxin-1 mutants used for the PRE studies of Fig. 2. SNN and SNC refer to the N- and C-terminal SNARE motifs of SNAP-25, respectively. (B) SNAP-25 and synaptobrevin mutants used for the PRE studies of Fig. 5-7. (C) Synaptobrevin and SNAP-25 mutants used for the ITC studies of Fig. 3, 8, the NMR analysis of Fig. 4 and the PRE studies of Fig. 5-7.

Figure S2. PRE analysis of Syt1 C_2AB binding to the C-terminal half of the SNARE complex. The graphs show bar diagrams of the PREs measured for the methyl groups indicated in the x axis in ^1H-^{13}C HMQC experiments performed with ^{15}N-^2H–ILV-$^{13}CH_3$–labeled Syt1 C_2AB and SNARE complex tagged with MTSL at residue 61 or 72 of syynaptobrevin (Syb61 and Syb72, respectively) (A, B), residue 239 of syntaxin-1 (Syx239) (C), or residue 65, 187 or 197 of SNAP-25 (SNN65, SNC187 and SNC197, respectively) (D-F). These measurements were used to generate Fig. 2C-H. The vertical dashed lines mark the separation between the C_2A and C_2B domains.

Figure S3. The $C_2B^{KA\text{-}Q}$ mutant is properly folded and binds Ca^{2+}. (A) Superposition of ^1H-^{15}N TROSY HSQC spectra of WT Syt1 C_2B domain (black contours) and the $C_2B^{KA\text{-}Q}$ mutant (red contours) in the presence of EDTA. (B) Superposition of ^1H-^{15}N TROSY HSQC spectra of the $C_2B^{KA\text{-}Q}$ mutant in the presence of 1 mM EDTA (black contours) or 1 mM Ca^{2+} (red contours).

Figure S4. The $C_2B^{KA\text{-}Q\text{-}LLQQ}$ mutant is properly folded and binds Ca^{2+}. (A) Superposition of ^1H-^{15}N TROSY HSQC spectra of the Syt1 $C_2B^{KA\text{-}Q}$ mutant (black contours) and the $C_2B^{KA\text{-}Q\text{-}LLQQ}$ mutant (red contours) in the

presence of EDTA. (B) Superposition of ^{1}H-^{15}N TROSY HSQC spectra of the $C_2B^{KA\text{-}Q\text{-}LLQQ}$ mutant in the presence of 1 mM EDTA (black contours) or 1 mM Ca^{2+} (red contours).

Figure S5. ITC analysis of interactions between $C_2B^{KA\text{-}Q}$ and SNARE^Q. The graphs show additional ITC data acquired with different preparations under conditions analogous to those of Fig. 3. The data in panel (A) were acquired in the Brunger lab and those of the other panels were acquired in the Rizo lab. The proteins used in each experiment and their concentrations in the cuvette or the syringe are indicated in each panel. The following groups of panels correspond to sets experiments performed on the same day with the same preparation: (B-C); (D-E); (F-G); and (H-J). The upper panels show the baseline- and singular-value-decomposition-corrected thermograms for the respective experiments. The circles in the lower panels are the integrated heats of injection, with the error bars representing estimated errors for these values.

Figure S6. ITC analysis of interactions of Cpx1(48-73) with SNARE^Q and $C_2B^{KA\text{-}Q}$. The panels show titrations of 10 μM SNARE^Q with 120 μM Cpx1(48-73) (A) or 30 μM Cpx1(48-73) with 460 μM $C_2B^{KA\text{-}Q}$ (B) monitored by ITC. The upper panels show the baseline- and singular-value-decomposition-corrected thermograms for the respective experiments. The circles in the lower panels are the integrated heats of injection, with the error bars representing estimated errors for these values. The curves represent the fits of the data to a single binding site "A + B <-> AB" model. The thermodynamic parameters derived for binding of Cpx1(48-73) to SNARE^Q are indicated in the bottom panel of (A).

Figure S7. Cross-peaks assignments of the ^{1}H-^{15}N HSQC spectrum of $C_2B^{KA\text{-}Q}$. Cross-peaks are labeled with the corresponding residue.

Figure S8. Analysis of potential tripartite Syt1-SNARE-complexin-1 complexes using PREs. The diagrams show the SNARE complex in light gray, complexin-1 in dark gray and two copies of the C_2B domain bound through the primary interface (pink) or the tripartite interface (lime green). The locations of residues that were mutated to cysteine for labeling with MTSL, namely residue D44 of synaptobrevin (red spheres) (A, C, E) and residue E27 of SNAP-25 (green spheres) (B, D, F) are indicated. Residues colored in blue indicate PREs smaller than 0.8 in ^{1}H-^{15}N TROSY HSQC experiments acquired with samples containing 20 μM ^{15}N,^{2}H-labeled $C_2B^{RKRR\text{-}EEQQ}$ (A,B) or ^{15}N,^{2}H-labeled $C_2B^{RK\text{-}EE}$ (D-F) in the presence of 110 μM MTSL-labeled SCCpx D44C (A, C), SCCpx E27C (B, D), $SC^{Q}Cpx$ D44C (E) or $SC^{Q}Cpx$ E27C (F) before and after reduction. The W404 side chain, which exhibits PREs smaller than 0.8 in all experiments, is shown as a blue stick model. The data used to generate these diagrams are shown in Fig. 5-6.

Figure S9. ITC analysis of interactions between $C_2B^{RK\text{-}EE}$ and $SNARE^{Q}$. The graphs show additional ITC data acquired with different preparations under conditions analogous to those of Fig. 8. The proteins used in each experiment and their concentrations in the cuvette or the syringe are indicated in each panel. The upper panels show the baseline- and singular-value-decomposition-corrected thermograms for the respective experiments. The circles in the lower panels are the integrated heats of injection, with the error bars representing estimated errors for these values.

Figure S10. Analysis of the localization of the Syt1 LLQQ and TGQQ mutants. (A) Representative immunoblot showing significant knockdown of Syt1, along with quantitative analysis of normalized Syt1 expression from cultures infected with sham lentivirus vs lentivirus carrying Syt1 knockdown construct (bars represents average ± SEM; two-tailed non-paired t test; n=6/group, **** denotes $p < 0.0001$). (B) Representative confocal images of different channels for synapsin (shown in green) and Syt1 (shown in blue), along with quantitative analysis of colocalization. Exogenously introduced (via lentivirus) WT Syt1

and Syt1 TGQQ and LLQQ mutants colocalize with synapsin to a similar extent as endogenously expressed Syt1. Each data point represents analysis of a single image from a unique coverslip (n=5/group, graph shows average ± SEM). Pearson´s correlation coefficients of the mutant rescues are compared with those of WT rescue with two-tailed Mann-Whitney test (all non-significant with $p>0.05$). Scale bars = 20 μM.

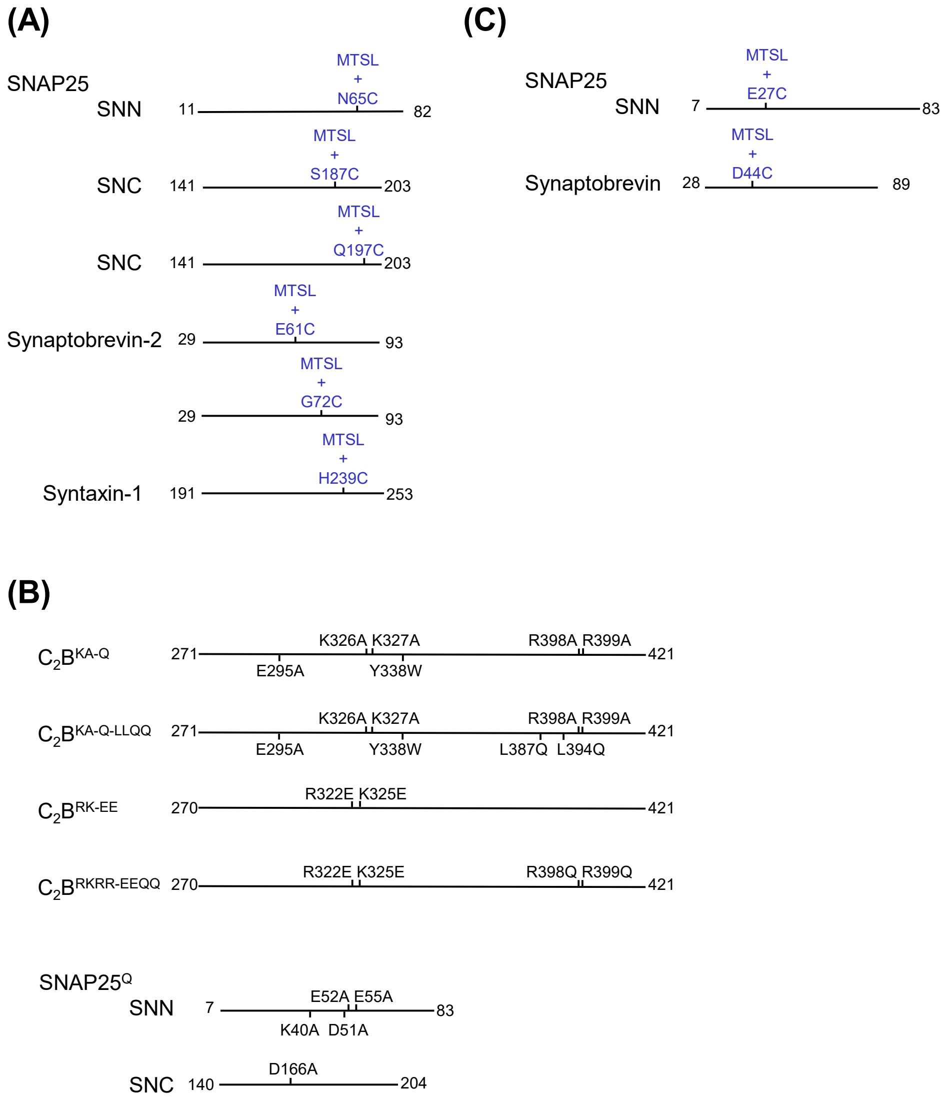

Figure S1
Jaczynska et al.

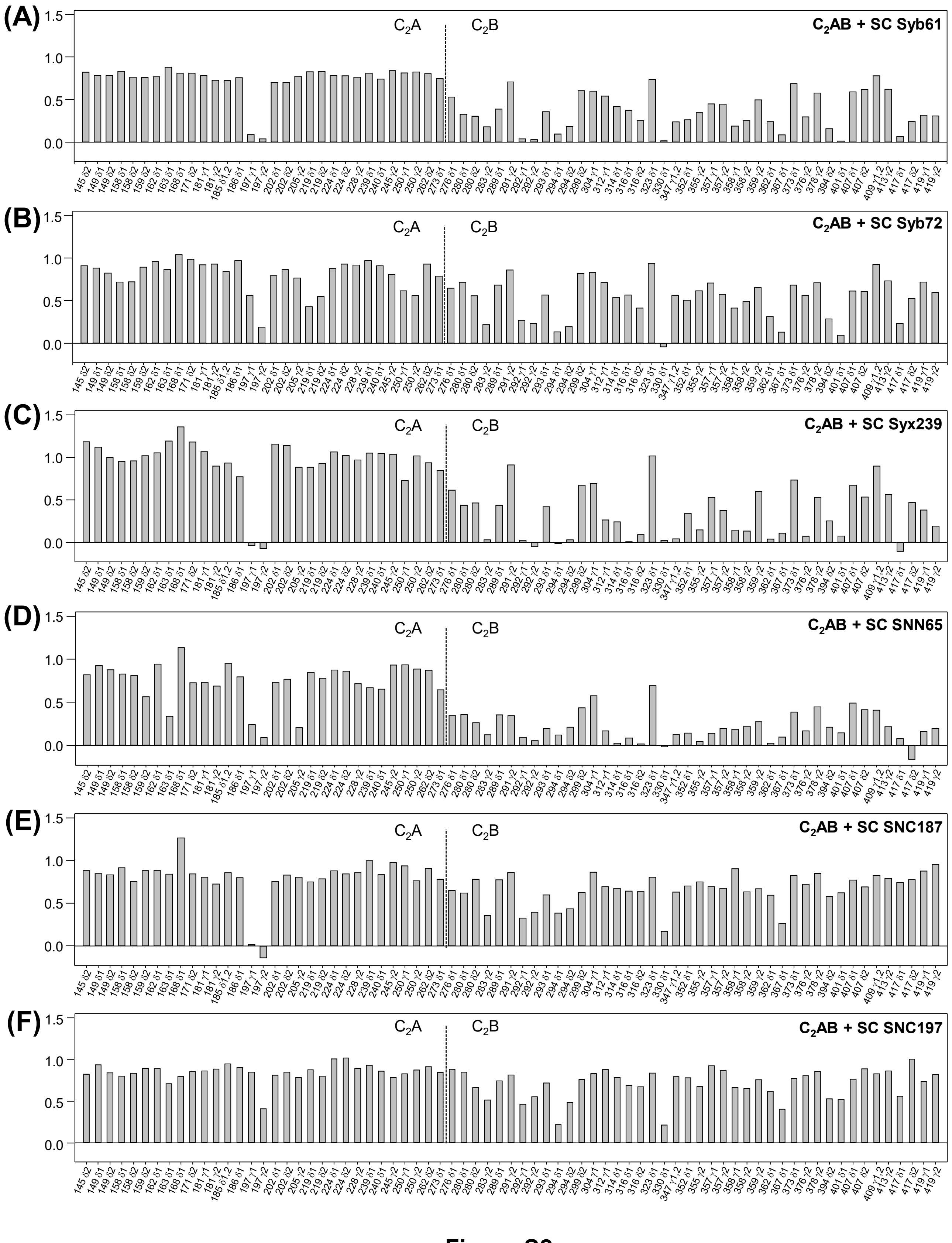

Figure S2
Jaczynska et al.

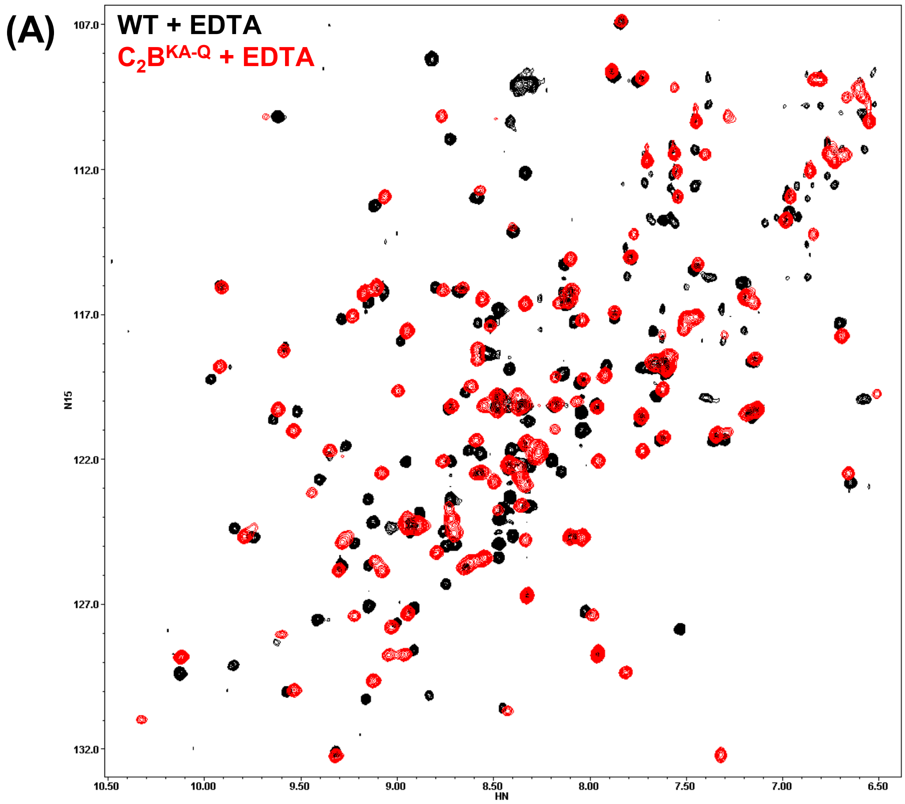

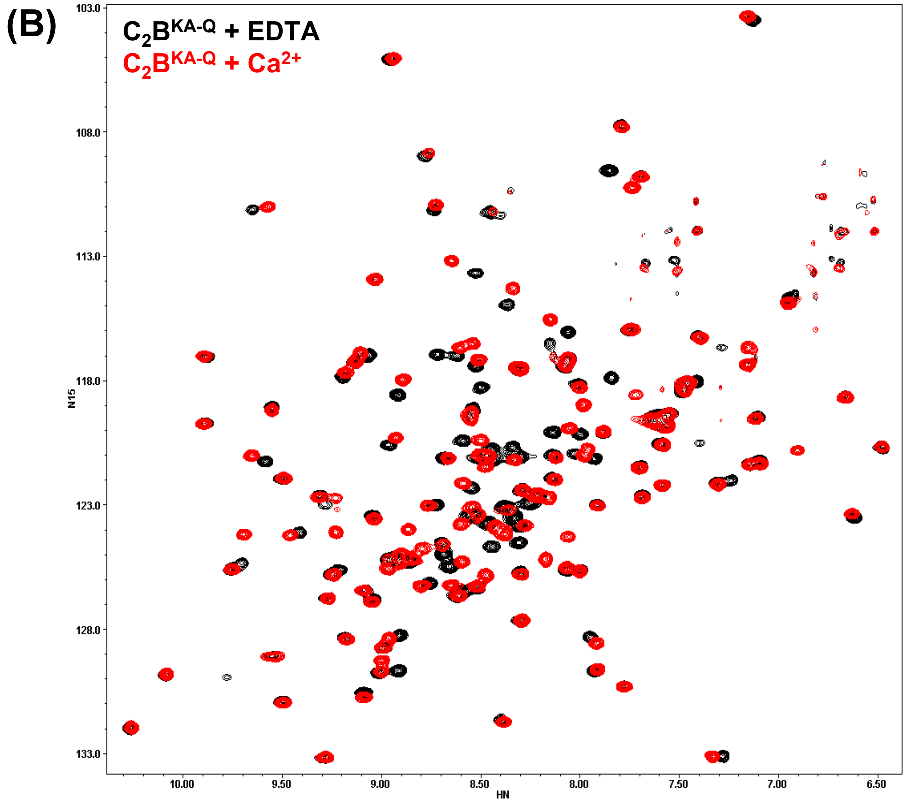

Figure S3
Jaczynska et al.

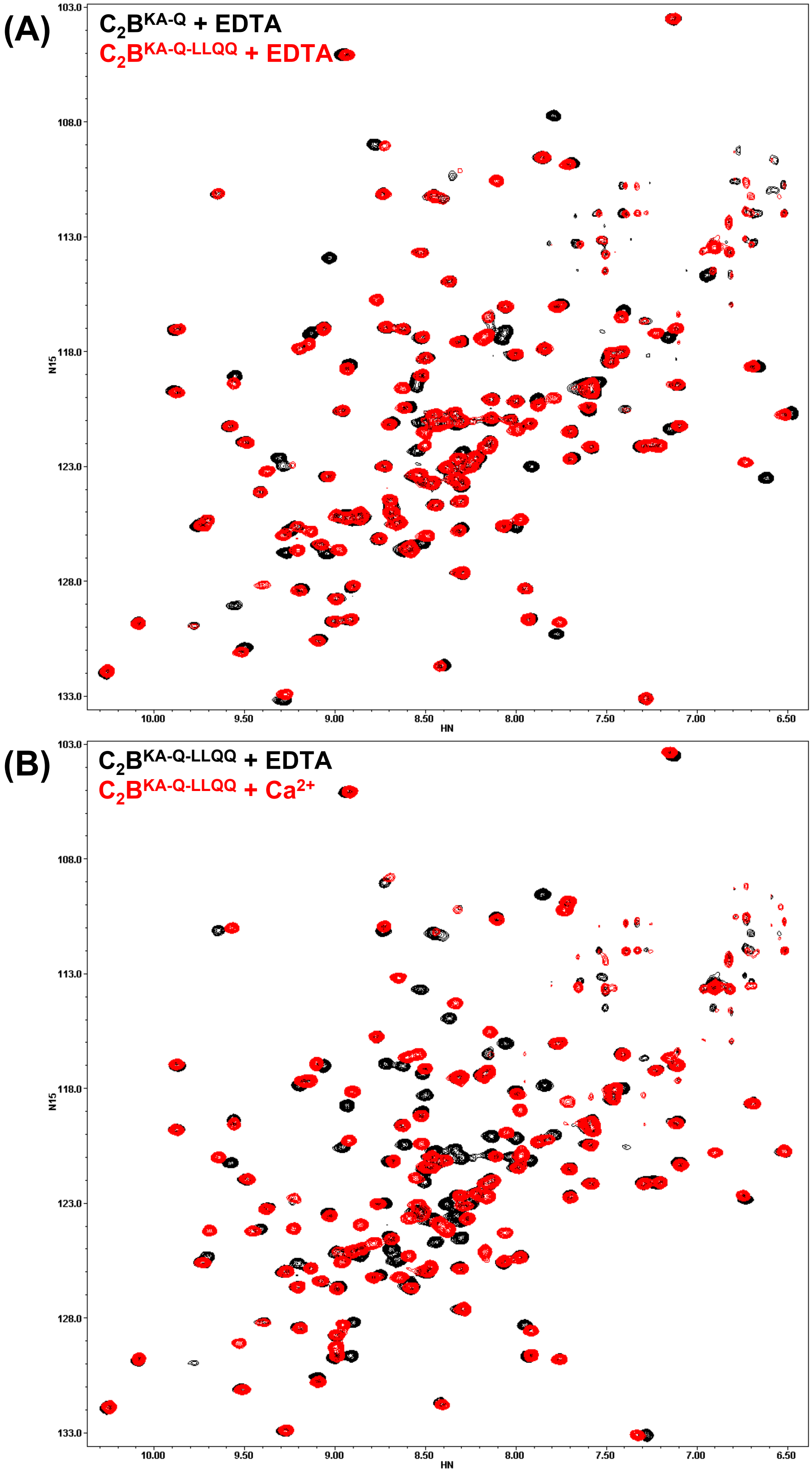

Figure S4
Jaczynska et al.

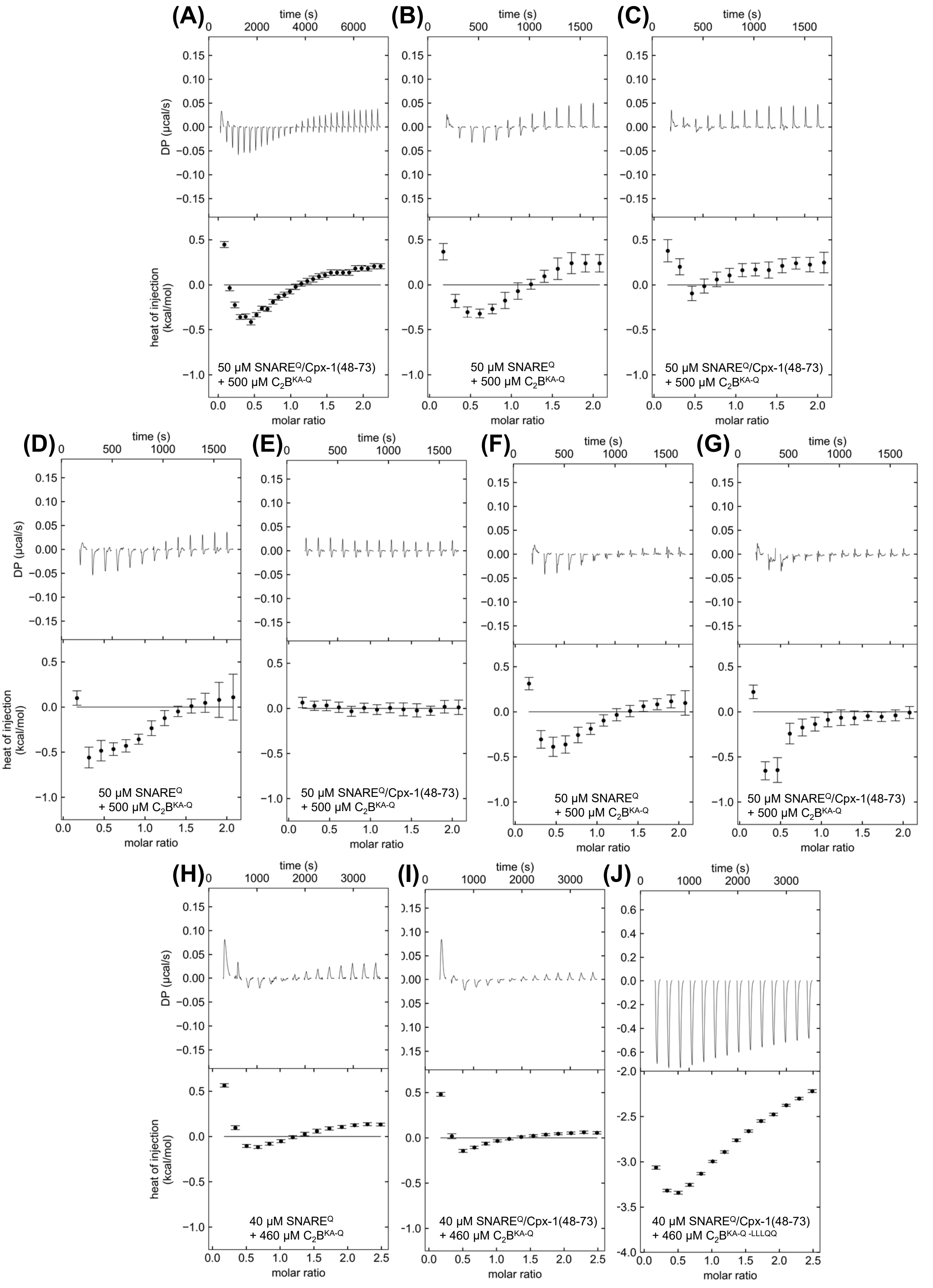

Figure S5
Jaczynska et al.

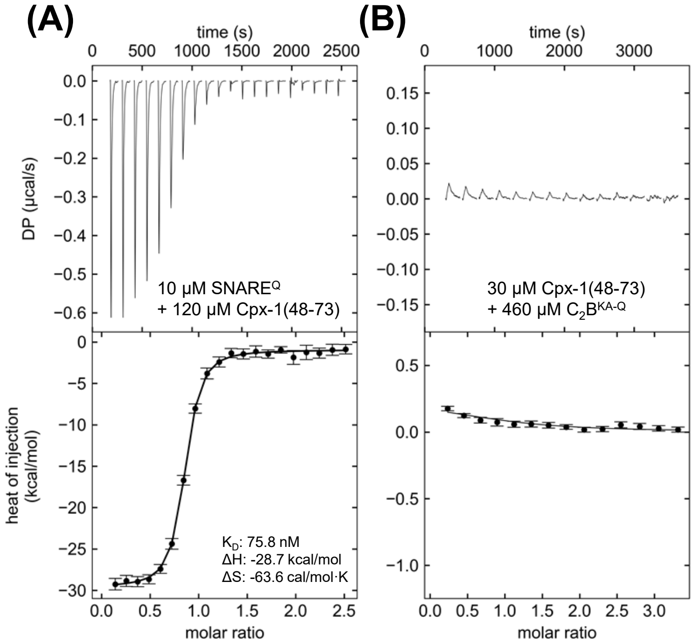

Figure S6
Jaczynska et al.

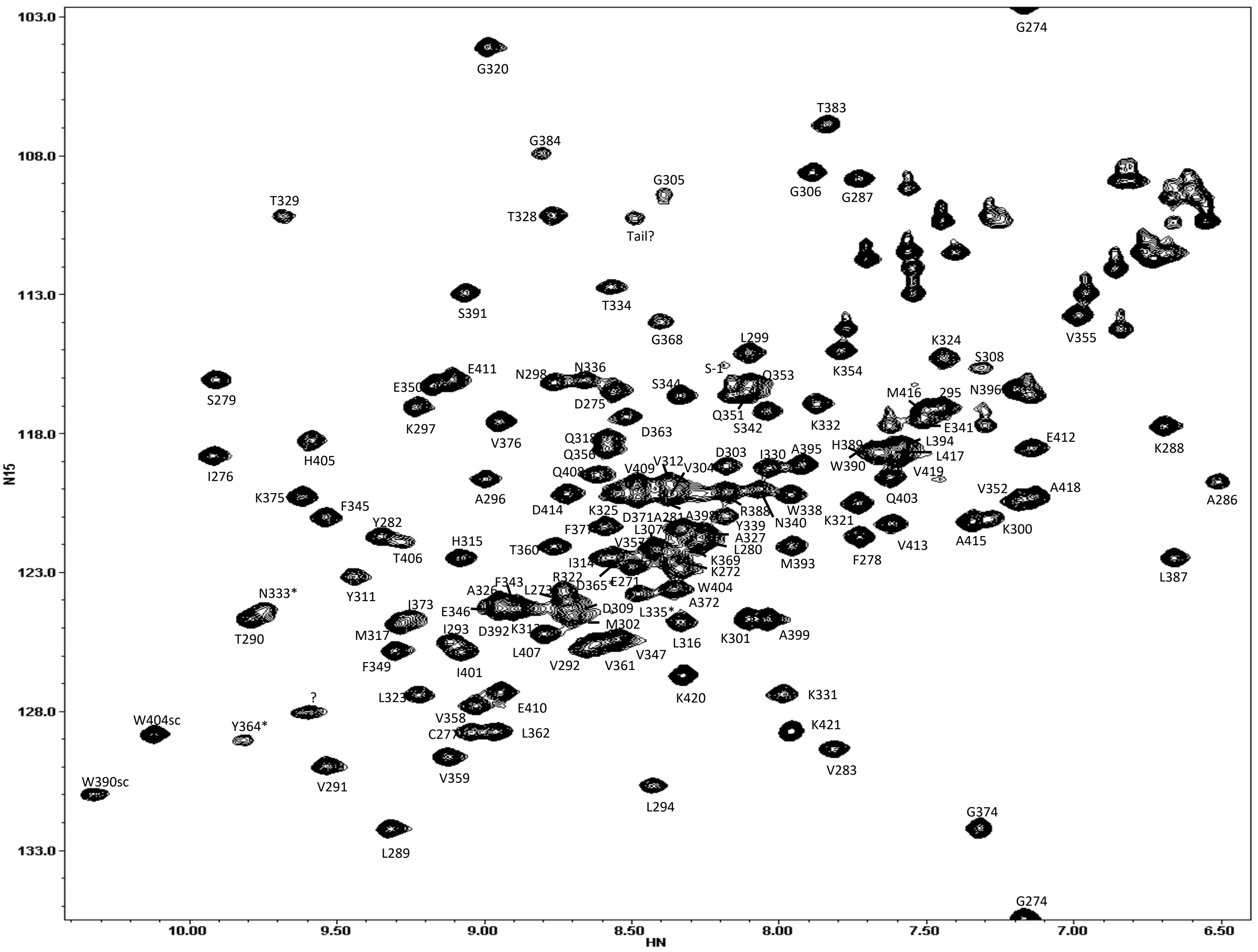

Figure S7

Jaczynska et al.

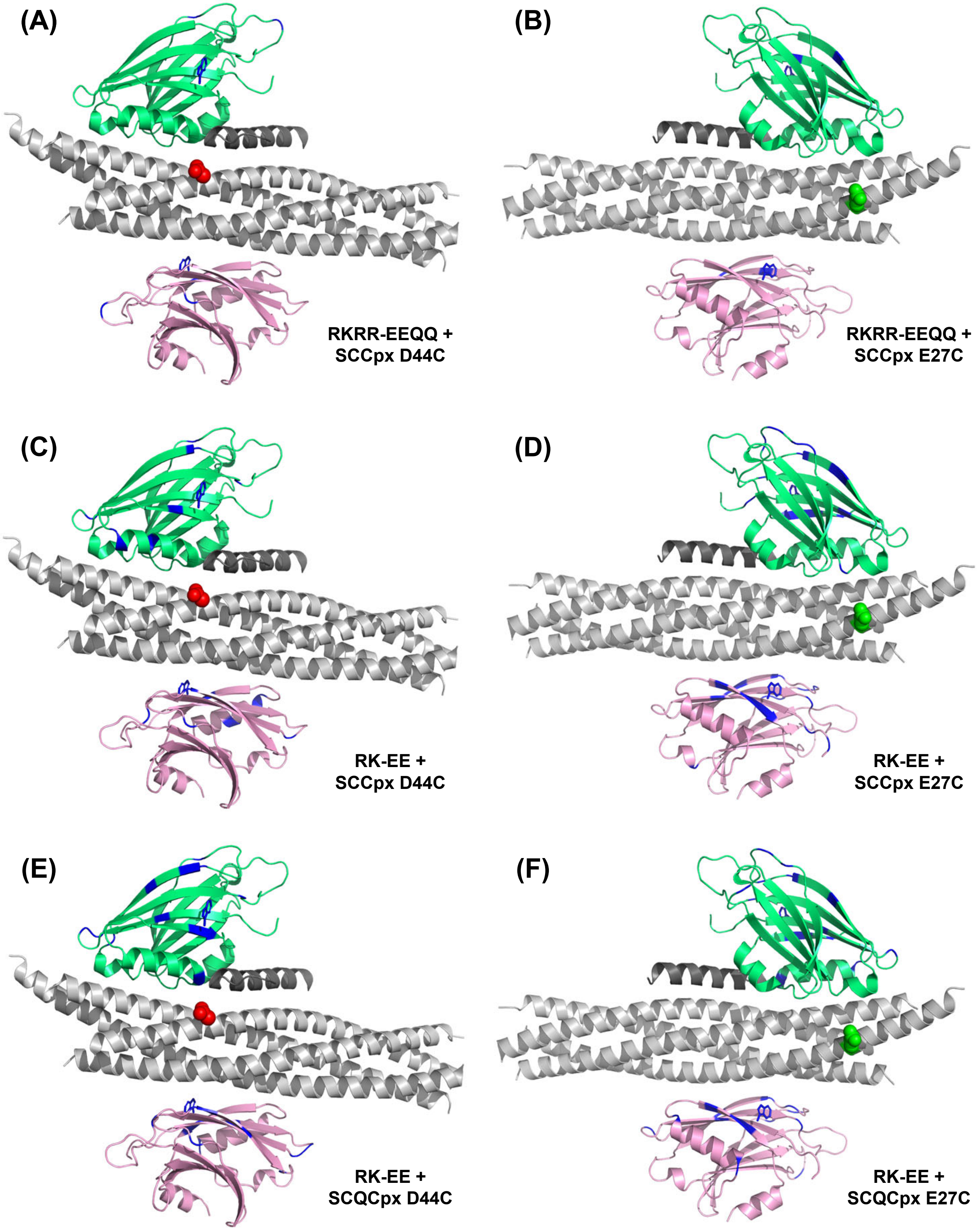

Figure S8
Jaczynska et al.

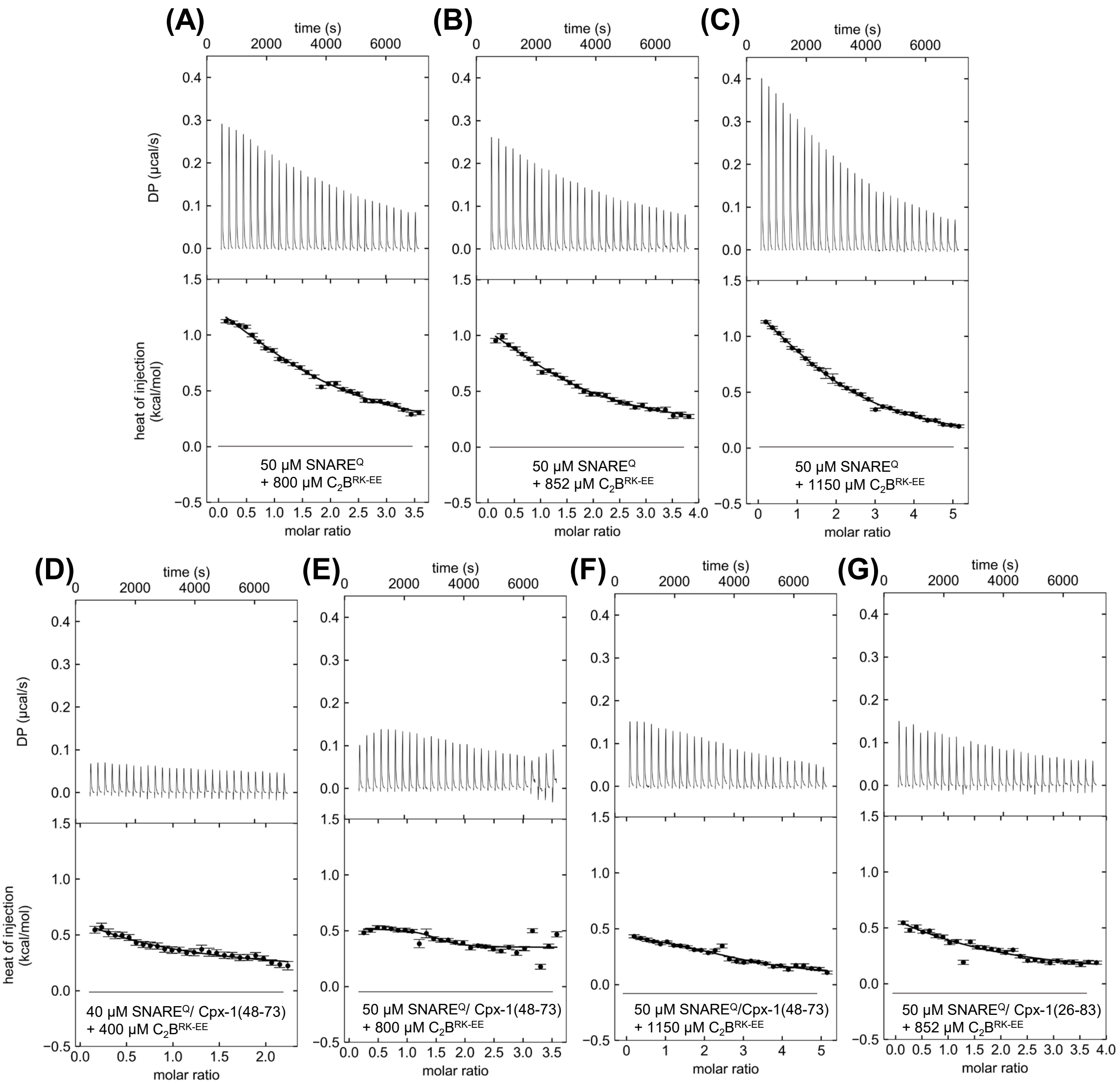

Figure S9
Jaczynska et al.

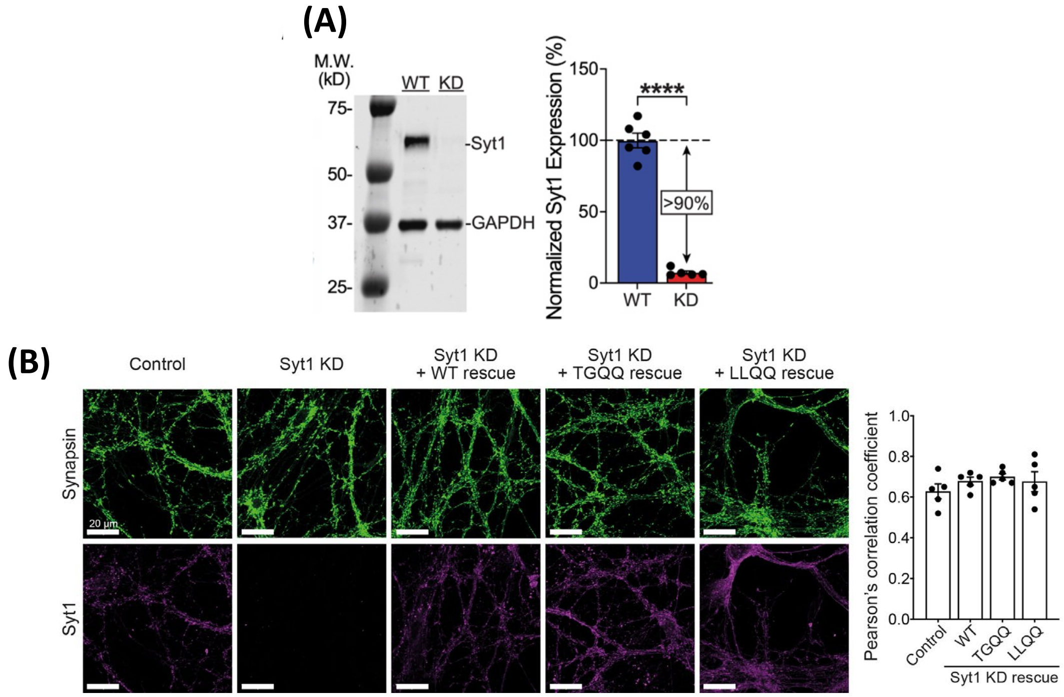

Figure S10
Jaczynska et al.
